# Free threonine in human breast milk is related to infant intestinal microbiota composition

**DOI:** 10.1007/s00726-021-03057-w

**Published:** 2021-09-03

**Authors:** Monika Riederer, Natascha Schweighofer, Slave Trajanoski, Claudia Stelzer, Miriam Zehentner, Bianca Fuchs-Neuhold, Karl Kashofer, Johannes A. Mayr, Marlies Hörmann-Wallner, Sandra Holasek, Moenie van der Kleyn

**Affiliations:** 1 Institute of Biomedical Science, University of Applied Sciences JOANNEUM, Graz, Austria; 2 Division of Endocrinology and Diabetology, Medical University Graz, Graz, Austria; 3 Core Facility Computational Bioanalytics, Center for Medical Research (ZMF), Medical University of Graz, Graz, Austria; 4 Institute of Dietetics and Nutrition, Health Perception Lab, University of Applied Sciences JOANNEUM, Graz, Austria; 5 Diagnostic and Research Institute of Pathology, Medical University of Graz, Auenbruggerpl. 2, 8036 Graz, Austria; 6 Department of Pediatrics, Salzburger Landeskliniken and Paracelsus Medical University, Salzburg, Austria; 7 Department of Pathophysiology, Medical University Graz, Graz, Austria; 8 Institute of Midwifery, University of Applied Sciences JOANNEUM, Graz, Austria

**Keywords:** Threonine, Free amino acids, Breast milk, Gammaproteobacteria, Human, Microbiota, Infant, Enterobacteriales

## Abstract

**Background:**

Accumulating evidence indicates that free amino acids (FAA) might be bioactive compounds with potential immunomodulatory capabilities. However, the FAA composition in human milk is still poorly characterized with respect to its correlation to maternal serum levels and its physiological significance for the infant. Studies addressing the relation of human milk FAA to the infants' intestinal microbiota are still missing.

**Methods:**

As part of a pilot study, maternal serum and breast milk FAA concentrations as well as infant intestinal microbiota (16S rRNA) were determined 2 months after birth. The study cohort consisted of 41 healthy mothers and their term delivered, healthy infants with normal birthweight. The relationship between maternal serum and milk FAA was determined by correlation analyses. Associations between (highly correlated) milk FAA and infant intestinal beta diversity were tested using PERMANOVA, LefSe and multivariate regression models adjusted for common confounders.

**Results:**

Seven breast milk FAA correlated significantly with serum concentrations. One of these, threonine showed a negative association with abundance of members of the class Gammaproteobacteria (*R*^2^adj = 17.1%, *p* = 0.006; *β*= − 0.441). In addition, on the level of families and genera, threonine explained 23.2% of variation of the relative abundance of Enterobacteriaceae (*R*^2^adj; *p* = 0.001; *β* = − 0.504) and 11.1% of variability in the abundance of Escherichia/Shigella (R^2^adj, *p* = 0.025; *β*  = − 0.368), when adjusted for confounders.

**Conclusion:**

Our study is the first to suggest potential interactions between breast milk FAA and infant gut microbiota composition during early lactation. The results might be indicative of a potential protective role of threonine against members of the Enterobacteriaceae family in breast-fed infants. Still, results are based on correlation analyses and larger cohorts are needed to support the findings and elucidate possible underlying mechanisms to assess the complex interplay between breast milk FAA and infant intestinal microbiota in detail.

**Supplementary Information:**

The online version contains supplementary material available at 10.1007/s00726-021-03057-w.

## Introduction

The FAA composition in human breast milk is still poorly characterized with respect to its physiological significance for infant development. As the contribution of FAA to the mass of total protein-bound AA in breast milk is quite low (approximately 5–10%), their contribution to nutritional aspects would be expected to be moderate (Zhang et al. 2013; van Sadelhoff et al. 2018). However, the proteolytic capacity of neonates is rather inefficient, and as compared to the FAA levels in maternal plasma, the concentration of distinct human milk FAA is up to 30 times higher in breast milk (van Sadelhoff et al. 2020).

Recent evidence indicates that FAA might be bioactive compounds (Roth, 2007; Ruth and Field 2013; Wu, 2013) with potential immunomodulatory capabilities. They have been described to be more rapidly absorbed, and to reach systemic circulation and peripheral organs faster than protein-bound amino acids (Carratù et al. 2003; Zhang et al. 2013; Schanler and Garza 1987; Koopman et al. 2009). In contrast to total AA (van Sadelhoff et al. 2018), several research groups reported a consistent and highly interesting AA-specific FAA pattern according to lactation stage. Importantly, this pattern was independent of ethnic or geographic factors, indicating FAA levels are tightly regulated and might play specific roles in the developing infant (Zhang et al. 2013; Garcia-Rodenas et al. 2016). In this context, the most abundant FAA glutamate and glutamine as well as glycine, serine and alanine show a significant increase during the first 3 months of lactation, whereas most other FAA decrease/or remain relatively stable (Zhang et al. 2013; Garcia-Rodenas et al. 2016; Yamawaki et al. 2005).

Remarkably, a few distinct species of breast milk FAA (glutamic acid and glutamine) have already been identified as important physiological mediators e.g. for the development of the immature infant gut and for the satiety status of the lactating infant (van Sadelhoff et al. 2020; Alison K. Ventura et al. 2012a, b). The underlying mechanisms responsible for those distinct FAA patterns during lactation have not been elucidated so far. Secreted proteases of the mammary gland cells and the regulated expression of AA transporters for the direct secretion of AA into breast milk have been suggested to be involved (Dallas 2012; Lin et al. 2018; Alemán et al. 2009; Dallas et al. 2015). Many of those mechanistic studies have been done in animals and to the best of our knowledge it is not entirely known how all the distinct FAA in blood and breast milk are related in humans.

Certain maternal and infant determinants of breast milk FAA have already been suggested in literature: Maternal body-mass index (BMI) and pre-pregnancy weight slightly influenced some FAA (Larnkjær et al. 2016; Jochum et al. 2006), and infant gender, gestational age (preterm), length and weight gain seemed to have an effect on breast milk FAA distribution (Baldeón et al. 2019). However, further information on maternal determinants as well as on the impact of the breast milk FAA profile on the healthy development of the infant is still sought. Therefore, our aim was to study the FAA profile in this highly regulated period—in blood and breast milk of healthy lactating mothers (of healthy, normal weight, term infants) in detail.

Regarding the proposed immune modifying properties of individual FAA, convincing evidence arose that the availability of specific AA (in particular glutamine, glutamate, and arginine, and eventually methionine, cysteine and threonine) are essential in the optimization of the immunological competence of the infants’ intestine (and adjacent immune cells) (Ruth and Field 2013; Field et al. 1994). In this context, the intestinal epithelium is not only responsible for nutrient absorption, but also plays a major role in protecting the infant from oral pathogens, in part by maintaining a healthy interaction with commensal bacteria (Ziegler et al. 2003). External microbes start to colonize the neonatal gut immediately after/during birth. The subsequent establishment of the infants´ microbiome is another important and highly susceptible factor in the development of immune (system) maturation. In the first months of life, gut microbial composition is highly dynamic, but stabilizes in childhood and provides the basis for a stable gut microbiota in adulthood. Disruption of early-life gut microbiota is suspected to affect metabolic programming (Cox et al. 2014) and to result in the development of obesity and other metabolic diseases during childhood (Mulligan and Friedman, 2017).

The composition and development of infant gut microbiota can be influenced by many factors, such as maternal obesity, smoking status, use of antibiotics, pre/term birth, mode of delivery and infant feeding mode (Vandenplas et al. 2020). Undisputedly, breast milk represents the most suitable nutritional resource for optimal infant growth, but it also harbors a set of bioactive components that drives the establishment and maintenance of early gut microbiota (as e.g. human milk oligosaccharides, proteins and FAA). Provision of different sources and amounts of dietary protein was already shown to influence gut microbiota and its metabolites, e.g. when comparing breast-fed with formula-fed infants (Kok et al. 2020). However, there is only limited data on how the different FAA in human milk relate to or even influence the composition of the infant's microbiota.

Therefore, we decided to investigate the relationship between maternal serum and milk FAA and to relate distinct breast milk FAA concentrations to infant intestinal microbiota at 2 months of life. At this time point, described FAA fluctuations and FAA intake is mainly influenced by breast and/or formula milk feeding, since it precedes the introduction of solid food.

## Methods

### Study protocol and cohort

**Fig. 1:**
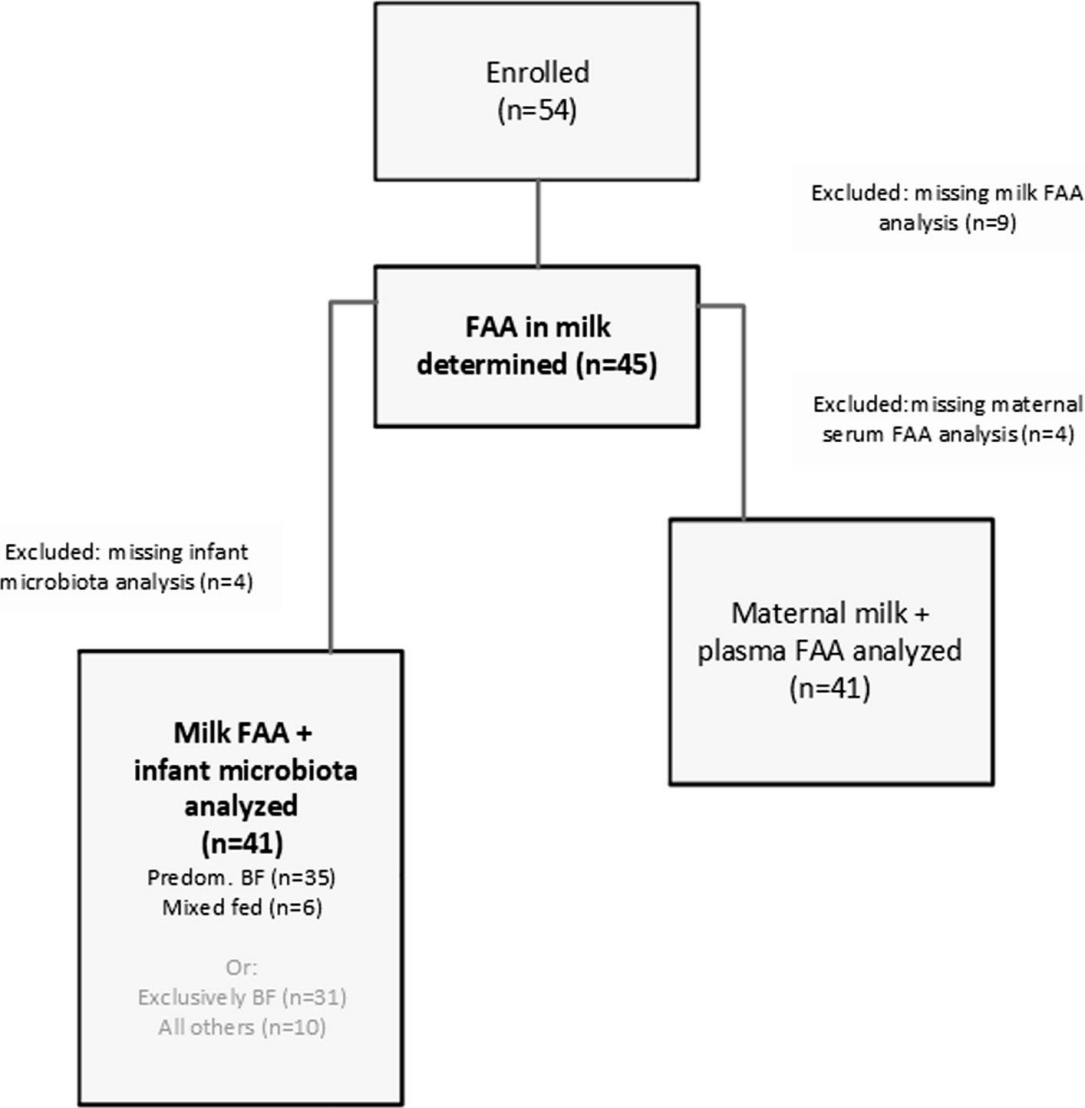
Cohort flow chart illustrating the inclusion and exclusion criteria applied to the dataset. The final cohort used for the analysis of maternal and infant parameters is shown at the left side (*n* = 41) and is described in detail in Table 1. Predom. BF = predominantly breast fed

The general study outline was described previously (Riederer et al. 2020). For this topic of the explorative study, a total of 45 lactating mothers were analyzed when their infants were aged between 6 and 8 weeks of life (out of 54 enrolled; in 9 samples AA were not determined in breast milk). For the main parameter “milk FAA”, sample count (n) was 45 (see descriptives of the study cohort in Table 1). Serum FAA and infant microbiota could not be determined in 4 samples each (resulting in 41 but not identical pairs each), as shown in Fig. 1.

**Table 1:** Baseline characteristics of mother and child pairs

Characteristics:	% or mean	SD	*n*
Maternal characteristics			
Austrian nationality	86.8		33
Maternal age at delivery (years)	32	3.4	41
Early pregnancy BMI (kg/m^2^)	22.5	2.8	41
Early pregnancy BMI category			
Low (BMI ≥ 18,5 to < 25 kg/m^2^)	82.9		34
High (BMI ≥ 25 to ≤ 30.5 kg/m^2^)	17.1		7
Gestational weight gain (kg)	14.7	4.3	37
Parity (%)			
Primipara	78.0		32
Multipara	22.0		9
Caesarean delivery (%)	36.6		15
Antibiotics use pre- or during birth (%)			
No	63.4		26
Yes	31.7		13
Breastfeeding practices at 2 mths (%)			
Exclusive breastfeeding	75,6		31
Breastfeeding plus < 30 ml formula	9,8		4
→ predominantly breastfed	85.4		35
Mixed fed	14.6		6
Infant characteristics			
Gestational age at delivery (days)	277	8	41
Sex of newborns (%)			
Female	48.8		20
Male	51.2		21
Birth weight (g)	3250.5	400.9	41
Percentile at birth*	42,9	27,5	41
Weight-for-Age Z-Score	-0,16	0,85	41
Infant characteristics at 2 months			
Age infant (days)	45	8	41
Weight (g)	4703.0	562.6	41
< P5*			1
> P85*			0
Weight gain from birth until 2 months (g)	1452.5	450.6	41

Metric variables are expressed in mean ± SD and categorical variables are expressed in percent

SD = standard deviation, *n*  = number of included participants, BMI = Body Mass Index

*WHO definition of normal weight-for-age: weight between 5th—85th percentile, according WHO growth charts

The main inclusion criteria were non-smoking women with an unobtrusive oral glucose tolerance test, an early-pregnancy BMI ≥ 18.5 kg/m^2^ to ≤ 30.5 kg/m^2^ (data assessed in early pregnancy up to pregnancy week 12), age between 18 and 50 years and their term born (> 37 + 0 weeks gestational age), normal weight (2500–4200 g), healthy infants. Women suffering from chronic health conditions, conditions influencing maternal feeding behavior as well as prematurity (delivery < 37 + 0 weeks gestational age) and multiple birth were excluded**.** BMI values for inclusion were calculated using a) self-reported early-pregnancy weight and b) maternal height (in centimeter) determined at site, in standing position with bare feet using the stadiometer Seca 213 (seca, Hamburg, Germany).

Maternal data were obtained from clinical records (Austrian mother and child booklet) as well as using questionnaires and personal interviews. Breastfeeding classification “exclusive”, “predominantly breastfed” versus “mixed feeders” was based on the anamnesis verified by an additional and detailed 24 h recall of the child's nutrition at two time points up to 2 months. Exclusive breastfeeding is defined by the only intake of breast milk including minor amounts of vitamins, medications, teas, or juices, corresponding to WHO's definition of breastfeeding (World Health Organization 2008; Labbok and Krasovec 1990). Predominantly breast-fed children (*n* = 35) ingested exclusively breast milk supplemented by water, and/or by up to a maximum of 30 ml formula; or by sporadic formula feeding from birth up to 2 months. Complementary food was not provided at all. The group of mixed feeders (e.g. breastfeeding complemented by more than 30 ml or regular feeding of formula) was small (*n* = 6). No infant was exclusively formula-fed.

### Ethics

The study was approved by the ethics committee of the Medical University of Graz (EC No 26–066 ex 13/14), and all participants gave written informed consent.

### Infant anthropometry

Data on weight and length were obtained through medical records or measured on site. At 2 months, infants’ length, weight and BC were measured by trained midwives during the visits in the laboratory. Length was obtained using a mobile measuring board (seca® 210, seca, Hamburg, Germany). BMI was calculated using the following equation including normalization to body height: BMI = body mass/body height, expressed as kg/m2 (Wells 2014). Infant weight-for-age (W/A), weight-for-length (W/L), and BMI-for-age were determined using the WHO Anthro Software (‘WHO | WHO Anthro (version 3.2.2, January 2011) and macros’, n.d.).

### Laboratory analyses of biochemical parameters

#### Sample collection

Maternal non-fasting serum samples were collected predominantly between 9 and 11 a.m., left to stand for 30 min at room temperature (according to the guidelines of the supplier, Greiner bio-one), centrifuged for 20 min at 2000 g, aliquoted after visual inspection and stored at – 80 °C (under constant temperature monitoring).

Breast milk was collected on site, under the supervision of midwives, between 9 and 11 a.m., using an electric breast pump (Harmony Breast Pump kit, Medela Inc., McHenry, IL) that dispensed milk into a 50 mL aseptic tube. When the infants were aged 2 months, all mothers were in the same lactating phase, producing mature milk. The sampling procedure was standardized as much as possible: Before sampling, women were asked to wipe the breast with sterile water pads. The samples were collected from one breast simultaneously to feeding at the other breast and foremilk and hindmilk were mixed; during minimal 15 min pumping and/or gaining minimal sample size of 40 ml of milk from one breast. Each collected sample was mixed by inverting and pipetting, aliquoted and immediately stored at − 80 °C.

#### FAA

FAA were determined in maternal serum and breast milk samples, collected when infants were 2 months old. Breast milk samples were centrifuged for 10.000×*g* for 5 min, and analyzed after removing the fat layer. The concentration of free AA was determined from serum or breast milk via ion exchange chromatography followed by postcolumn derivatization with ninhydrin using the Biochrom 30 + AA Analyzer Physiological System (Biochrom Ltd., Cambridge, UK). Serum samples were deproteinized by mixing 100 µl serum with 100 µl Seraprep (Pickering Laboratories, Mountain View, CA, U.S.A.) and adding 10 µl of 210 µmol/l norleucine as internal standard. After incubation for 30 min on ice, the samples were centrifuged at 10,000×*g* for 5 min. The supernatant was collected and filtered through a 0.2 µm centrifugal filter (Laborservice Onken, Gründau-Breitenborn, Germany). The flow-through was collected and 30 µl were loaded on the AA analyzer. For quantification an AA standard mixture (Laborservice Onken, Gründau-Breitenborn, Germany) was analyzed after at least every 20th sample. For quality control the analyzing laboratory takes part in the ERNDIM EQA scheme for AA (https://www.erndim.org).

#### Microbiota

Bacterial DNA from infant gut microbiota was isolated from pea-sized human stool samples. Samples (approximately 1 g) were collected in stool sample containers (including 1 ml RNAlater) and immediately frozen and stored at − 20 °C until DNA isolation and sequencing analysis. DNA was extracted using the Magnapure Bacterial DNA Kit following the manufacturers recommendations. The variable V4 region of the bacterial 16S rRNA gene was amplified with the 16S Basic Mastermix (Molzym GmbH, Bremen, Germany) using primers 16s_515_S3_fwd—TGCCAGCAGCCGCGGTAA and 16s_806_S2_rev – GGACTACCAGGGTATCTAAT. In a second round of PCR, Ion Torrent specific adaptor sequences and sample barcodes were added. Sequencing reactions were performed on Ion Torrent PGM using the Ion 400BP Sequencing Kit running for 1082 flows (all reagents from Thermo Fisher Scientific, MA, USA), using Ion Torrent 318 chips.

Quantitative Insights into Microbial Ecology 1 and 2 (QIIME), a bioinformatic pipeline integrated in the open source web-based platform Galaxy hosted on the MedBioNode HPC cluster of the Medical University Graz, Austria, was used to analyze the final sequence files. A total of 2 725 178 ( mean 60,559, sd 23,703) raw sequence reads were quality-filtered, de-noised, de-replicated, merged and checked for chimeras using DADA2 denoise-pyro pipeline (Callahan et al. 2016) with optimized parameters: p-trunc-len: 290, p-trim-left: 18 and p-max-ee: 3.0 as implemented in QIIME2 2019.7 microbiome bioinformatics platform (Bolyen et al. 2019). Taxonomic assignment of the DADA2 representative sequence set was provided with the QIIME2 sklearn-based classifier against SILVA rRNA database Release 132 at 99% identity (Quast et al. 2012). Phylogenetic tree was created with FastTree on Mafft aligned representative sequences (Price et al. 2010; Katoh and Standley 2013).

### Statistics

Descriptive data are presented as mean (+ / − standard deviation [SD]) for continuous variables and count (percentage) for categorical variables. The assumption of normal distribution was proven with Shapiro–Wilk and Kolmogorov–Smirnov tests and Q–Q plots. In case of skewed distributions, parameters were either log transformed or analyzed using non-parametric methods.

*FAA in human milk* (absolute and relative amounts) were correlated with serum FAA, using the Pearson or Spearman correlation coefficient, depending on data distribution. For the main parameter “milk FAA”, sample count (n) was 45. As serum FAA and infant microbiota could not be determined in 4 samples (this results in 41 different pairs each) and some single measurements of parameters had to be excluded (< limit of detection), n may vary depending on the parameters analyzed and on the parameter combinations (see Table 2, column “n missing”).

FAA values were classified into 2 groups “high” and “low” according to the distribution in the current dataset, with "low" extending to the mean or median of the respective FAA.

#### Microbiota

Further downstream statistical data analysis of microbiota composition including alpha and beta diversity was conducted with the R 4.0.3 program for statistical computing (https://www.R-project.org).

Alpha- and Beta Diversity: To explore the alpha diversity, alpha rarefaction plotting was used as a function of sampling depth and to determine whether the richness of the samples is fully noticed. Rarefaction cut off was set to 4000 reads. Species richness was defined by the number of determined features in every sample, and for sample diversity the Shannon Index and Faith phylogenetic Index (PD) were calculated (Keylock 2005; Faith 1992). To analyze microbial beta diversity weighted UniFrac distances were calculated (Lozupone and Knight 2005; Lozupone et al. 2007).

#### Visualization

Alpha-diversity comparisons were drawn as box plots. Principal coordinate analysis (PCoA) plots were created using weighted UniFrac distances and R package to visualize beta diversity (Vázquez-Baeza et al. 2013). Relative abundance of features was presented in stacked bar charts (representing 100%), using data filtered for bacterial entities with abundances above zero in at least 10 samples.

The associations of distinct FAA with infant microbiota composition was evaluated using permutational multivariate analysis of variance (PERMANOVA, Adonis) with weighted Unifrac distances as implemented in the vegan package (‘adonis function | R Documentation [Internet].’, 2021). A *p* value < 0.05 was considered as statistically significant. (Anderson 2001) Adjustment of the p value for multiple testing was performed (False discovery rate, FDR). For Linear discriminant effect size (LefSe), FAA values were classified into 2 groups “high” and “low” according to the distribution in the current dataset, with “low” extending to the mean or median of the respective FAA. Data were filtered for bacterial entities with abundances above zero in at least 10 samples. LefSe was performed to detect statistically different features between groups with high or low threonine concentration taking into account biological compatibility and effect relevance. (‘LefSe’, n.d.) Values were adjusted for FDR. Since LefSe provides the possibility to address confounders (by including subclasses in the analysis), we readily included the variable “birth mode” as a subclass (which is known to significantly influence infant microbiota). First LefSe applies a Kruskal–Wallis-Test followed by pairwise Wilcoxon Tests on subclasses and defines a Linear Discriminant Analysis (LDA) score, which is a measurement of effect size. All analyses were based on the methods described by Segata et al. (Segata et al. 2011). LefSe was performed on a Galaxy server provided by the Huttenhower lab (available from http://huttenhower.sph.harvard.edu/galaxy/) and used with the following settings: Alpha value for the factorial Kruskal–Wallis-Test = 0.01 (Threonine high versus low group), alpha value for pairwise Wilcoxon test between subclasses = 0.05 (birth mode vaginal versus section), threshold on the logarithmic LDA score for discriminative features = 2.0, all-against-all multiclass analysis. P-values of < 0.05 were considered significant in Kruskal–Wallis and pairwise Wilcoxon test. LDA score was log10 transformed and ≥ 2.0 was considered relevant. (Segata et al. 2011).

#### Regression models

Using standard uni- and multivariable linear regression models using FAA as independent variables, factors influencing the outcomes of infant bacterial composition were identified for the full data set and for subgroup data sets. All assumptions for the standard linear regression model were verified in advance. Residuals were checked for approximate normal distribution (Q–Q-Plot) and independence and problems of multicollinearity and heteroscedasticity were considered with formal tests and graphical methods. The models were adjusted for influencing variables with a strong background in literature (by inclusion of all covariates in a regression model and saving the standardized residuals as adjusted variable), described to affect FAA and microbial composition (GWG, parity, infant sex, mode of birth, feeding mode)—always considering the maximum number of total variables within the model to ensure the validity of the regression model. In addition, a complete model including all confounders was calculated, demonstrating the predictive contribution of the FAA on top of potential confounders.

## Results

### Descriptives of the study cohort

For the analysis of breast milk FAA, our cohort included 45 women. The cohort represented a homogeneous group of healthy lactating women and their normal birth weight (between 2.500 and 4.200 g) term infants, with stringent exclusion criteria as described in materials and methods. In Table 1, our cohort used for association of breast milk FAA and infant microbiota (*n* = 41 mother infant pairs) is shown.

### Descriptives FAA

FAA were determined in maternal serum and breast milk samples, when the infant was approximately 2 months old. As shown in Table 2, at 2 months, the most abundant maternal **serum FAA** were glutamine (581,5 µmol/L), alanine, glycine, proline, valine, lysine, serine, taurine, leucine, threonine, ornithine, arginine, histidine (104,4 µmol/L), in decreasing order of concentration. In **breast milk**, the most prominent FAA were glutamic acid (1609 µmol/L), glutamine, taurine, alanine, serine, glycine, threonine and aspartic acid (71 µmol/L) in decreasing order of concentration.

**Table 2:**
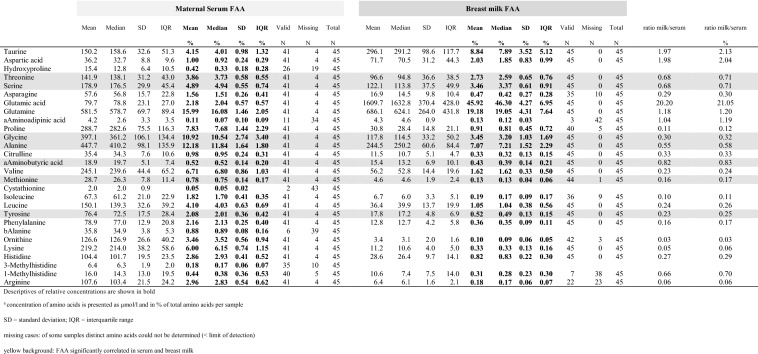
Absolute and relative (%) FAA concentrations in maternal serum and breast milk (at 2 months of age)

When the **relative contribution** of the FAA was addressed (Table 2, bold values), glutamine, alanine and glycine were the most prominent AAs in maternal serum at more than 10% each. In human milk glutamate (comprising as much as 45.9%), followed by glutamine and taurine were the most prominent molecular species. Although the sum of all FAA was similar in serum and in milk (3698 µmol/L versus 3594.9 µmol/L; * t* test: *p* = 0.352) the concentration of distinct amino acids differed significantly. As depicted in the last row of Table 2 (ratio milk/serum), in comparison to serum concentrations, glutamic acid was 20 times higher in milk, taurine and aspartic acid were twofold higher in milk, whereas ornithine and lysine were very low (10% of serum) in milk.

### Correlation analyses maternal serum versus breast milk FFA

To elucidate the relationship between the respective FAA (concentrations) in maternal serum and breast milk, correlation analyses were performed. After selection of FAA a) which were present in both matrices (serum and milk) and b) with valid data in more than 20 samples (each), correlation analyses were performed with the 22 remaining FAA. For purposes of better comparability, from now on—only relative values were used for comparison of FAA (= % of total FAA). As shown in Table 3, seven FAA exhibited strong positive correlation between serum and breast milk samples (e.g. threonine in milk with threonine in serum) after Bonferroni correction for multiple testing. (*r* > 0.5; *n* > 20).

**Table 3:** Significant correlations between respective FAA in serum and breast milk (e.g. serum threonine with human milk threonine)

FAA (%):	*r*	*p*	*n*	*p*_BFcorr_
Alpha-Aminobutyrate	0.807	0.00000000019	41	0.00000000419
Threonine (S)	0.795	0.00000000056	41	0.00000001225
Methionine	0.686	0.00000104585	40	0.00002300872
Alanine	0.605	0.00002823543	41	0.00062117948
Serine	0.59	0.00004347669	41	0.00095648723
Tyrosine	0.541	0.00025852339	41	0.00568751450
Glycine	0.528	0.00039185934	41	0.00862090547

Table shows significant results of correlation analysis of distinct amino acids with *r* > 0.5 and *n* > 20; selection of correlation coefficient (r) according to the distribution of FAA: (S) Spearman coefficient for not normally distributed values (as threonine); Pearson correlation coefficient for all other variables; p_BFcorr_: Bonferroni-corrected *p*-values.

As shown in Table 3, alpha-amino butyric acid, threonine, methionine and alanine exhibited the highest and most significant correlation between serum and breast milk. Those human milk FAA all belong to the group of FAA exhibiting higher concentrations in maternal serum than in breast milk (ratio: 0.017–0.083, Table 2).

Additionally, a significant correlation between two different amino acids—alpha-amino butyric acid (in serum) and alanine (in breast milk) was identified with *r* = − 0.574. The sum of FAA in blood and breast milk, however, did not show significant correlation (Pearson *r* = 0.295, *p* = 0.069; data not shown). The detailed results of the correlation analyses are depicted in online resource 2.

### Microbiota

Since human milk FAA are described as functional amino acids that may be important for the development of infant immunity, which often originates in the gut, their influence on the composition of the infant's intestinal microbiota was investigated. Those seven FAA that correlate highly between maternal serum and breast milk (Table 3) seem to be particularly relevant in this aspect, as they may represent maternal determinants of the infant’s microbiome which might be influenced e.g. by life style and can be easily monitored.

Infant microbiota composition of our cohort is shown in online resource 3ab (an overview of predominant taxa in 3a and the full list of taxa in 3b). In brief, the most abundant phyla were Firmicutes, Actinobacteria, Proteobacteria and Bacteroidetes, in decreasing order of relative abundance (36%, 26%, 22%, 16%). The most abundant classes were Actinobacteria, Gammaproteobacteria, Bacteroidia, Clostridia and Bacilli in decreasing order of relative abundance (25%, 21%, 15%, 15%, 13%). The most abundant orders were Bifidobacteriales, Enterobacteriales, Bacteroidales, Clostridiales and Lactobacillales in decreasing order of relative abundance (24%, 19%, 16%, 15%, 12%). With respect to the family level, Bifidobacteriaceae were followed by Enterobacteriaceae, Bacteroidaceae and Clostridiaceae (24%, 19%, 12% 11%). On the level of genera, Bifidobacterium, Escherichia/Shigella, Bacteroides and Clostridium were most prominent (24%, 13%, 12%, 11%).

In order to determine the microbiome variation attributable to the seven individual human milk FAA (identified above), we conducted a permutational analysis of variance (PERMANOVA; Adonis 1) with continuous variables. As two of the FAA (serine and alpha-aminobutyrate) showed significant intercorrelation with other FAA, they were excluded from the analyses. Notably, adonis analysis identified one milk FAA, threonine, potentially affecting the composition of the microbiota (*R*^2^ = 7.1%, p adj = 0.045), (Table.4).

**Table 4:** Permanova (Adonis 1) analysis output of microbiota composition in relation to 5 human milk FAA

	FAA (%)	Df	Sums Of Sqs	Mean Sqs	*F*	*R*^2^	*p*	p _adj FDR_
	LOG_Threonine	1	0.22161501	0.22161501	2.99041646	0.07121664	0.009	0.045
Residuals		39	2.89022794	0.07410841		0.92878336		
Total		40	3.11184294			1		

Only significant results are shown. Df: degrees of freedom, Sqs (Squares), *R*^2^ (coefficient of determination), *F* value, *p* (unadjusted *p* value); *p*_adj FDR_ (*p* value adjusted for FDR).

### Alpha diversity

Alpha-diversity measures (richness, Shannon, Faiths phylogenetic diversity (PD)) were not significantly correlated with threonine continuous values (data not shown). For further analysis and purposes of visualization, the threonine values were each categorized into two classes (0 low, 1 high) according to their median value (median:2.69%) in the current data set. Using those classes, differences in alpha – and beta – diversity were analyzed: In infants obtaining breast milk with high threonine concentration, richness was significantly increased (Wilcoxon; *p* = 0.03188). Faiths phylogenetic diversity (PD) and Shannon index whereas were not significantly different (for visualization see Online resource 4 a–c).

### Beta Diversity

**Fig. 2:**
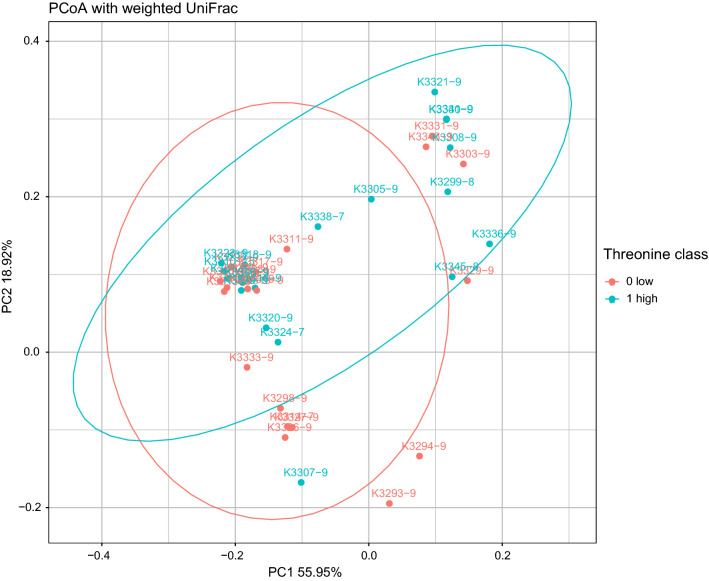
PCoA plot of the infant gut microbiota based on the weighted UniFrac metric. Red points represent microbiota of infants that received low threonine in breast mik (group 0). Blue points represent microbiota of infants that received higher amounts of threonine in breast milk (group 1), (group threshold: 2.69%)

Beta diversity was further addressed using principal component analysis of weighted UniFrac distances, where threonine subgroups (0 low,1 high) were indicated in red and blue, respectively (Fig. 2).

**Fig. 3:**
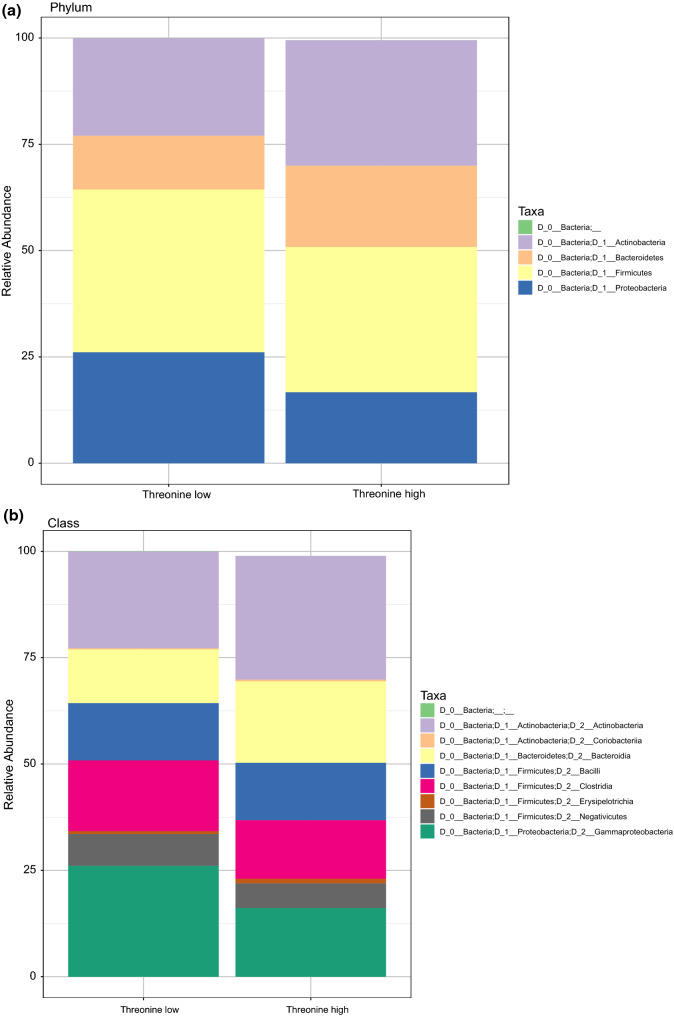

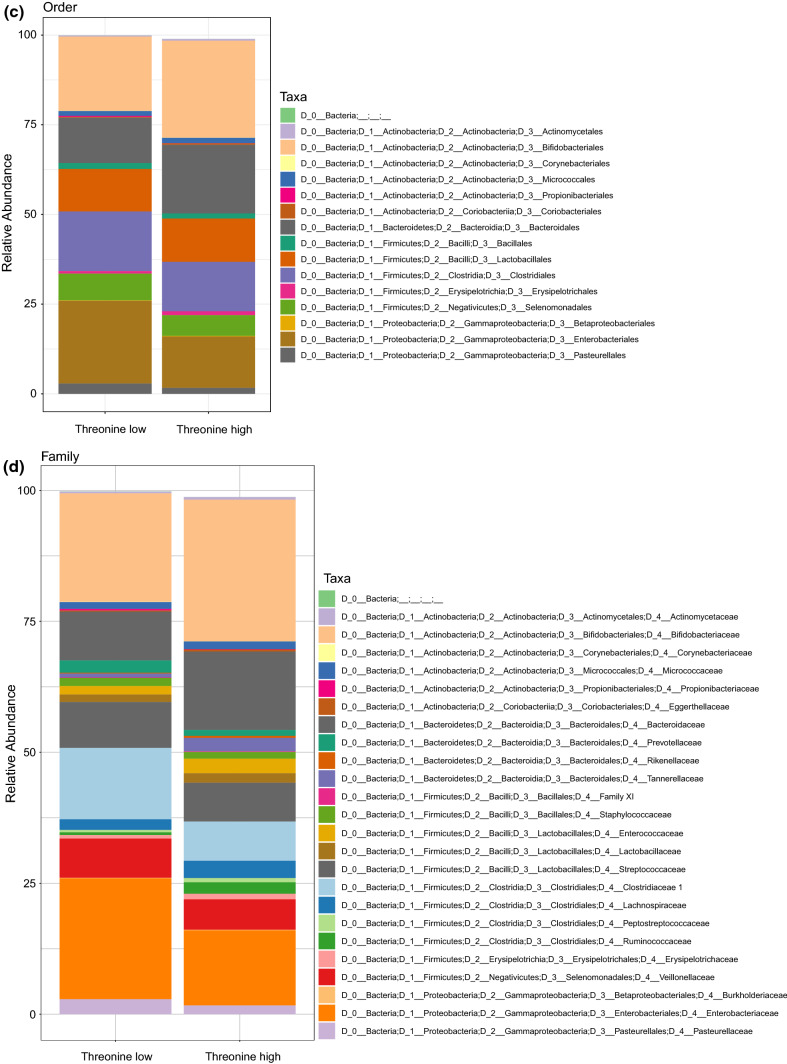
Stacked bar charts indicating differential composition of taxonomic groups between threonine groups. Stacked bar chart of microbial abundances of infant microbiota samples according to breast milk threonine group classification. Results are shown at the phylum (**a**), class (**b**), order (**c**) and family level (**d**) and are calculated as a percentage of total 16S rRNA reads within each group (filtered for entities present in more than 10 samples)

In order to visualize potential differences in distribution of taxonomic groups between the amino acid classes, stacked bar charts were generated on the level of phyla, class and order (Fig. 3). The bar charts present the most prominent bacterial groups (present in more than 10 samples).

**Fig. 4:**
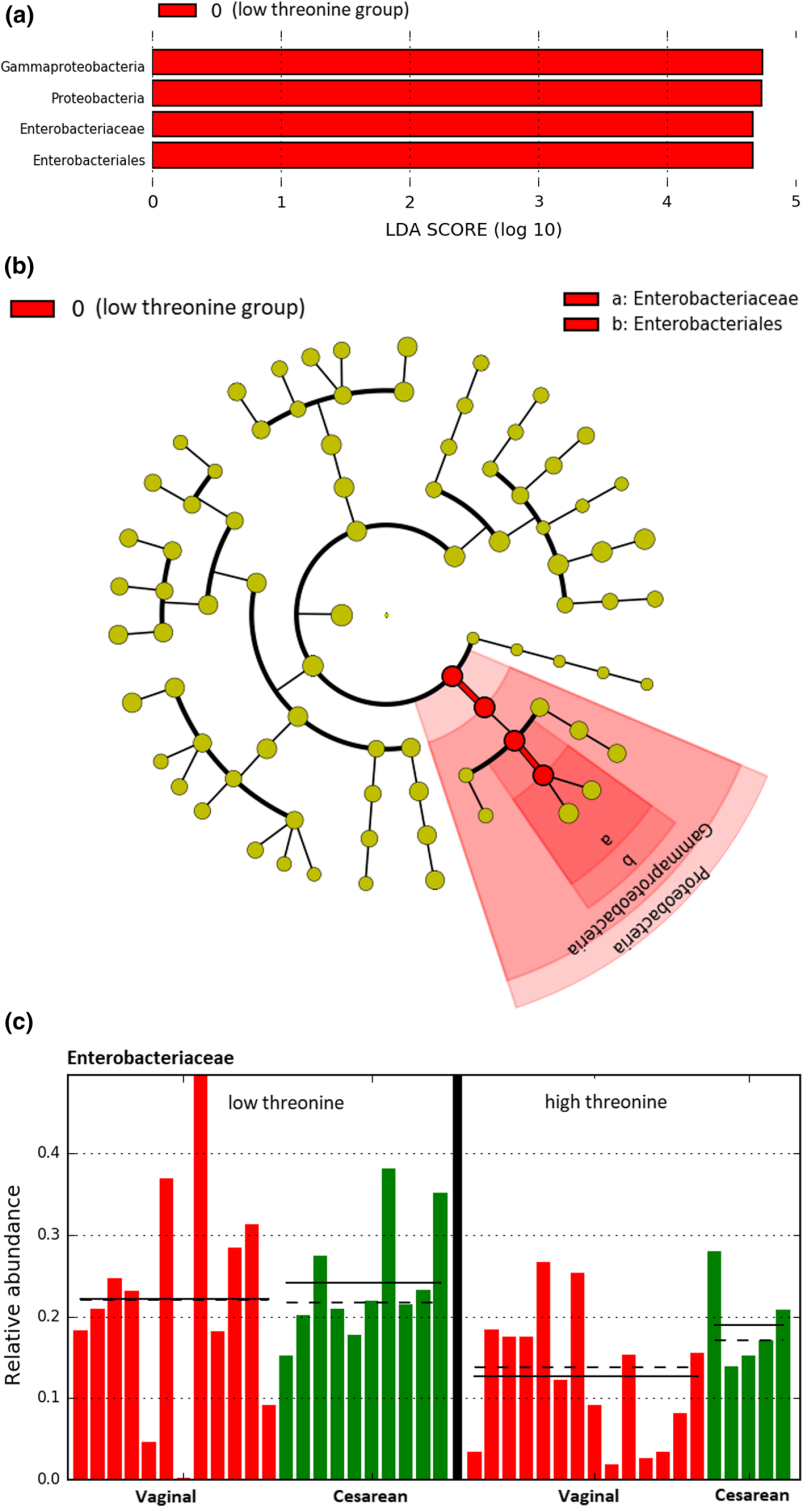
**a–c** LEfSe analysis identified microbial taxa enriched in infants receiving low threonine in breast milk (0). **a** Lefse result showing the taxonomic groups discriminating the microbiota composition of infants fed milk with high versus low FAA threonine. Bar graph showing LDA scores for taxa enriched in the low threonine group (0). (*p* < 0.05, LDA score > 2). **b** Cladogram generated by LEfSe indicating differences (described at a) at phylum, class, family and genus levels between the two groups (threonine high and low). The central dot represents the kingdom (Bacteria), and each successive circle represents the next phylogenetic level. Regions in red indicate taxa enriched in the low threonine group (0). **c** On the family level, Enterobacteriaceae show different abundance in infants receiving different threonine concentrations, irrespective of birth mode. The graph shows the relative distribution of Enterobacteriaceae in infant microbiota samples, as a result of LEfSe analysis with threonine as class and birth mode as subclass. (*p* < 0.05, LDA score > 2).

To further examine the variation in beta diversity attributable to the FAA, we used the LDA- effect size tool, LEfSe, for identification of taxonomic groups that were most informative for separating infants with high vs. low threonine levels (Fig. 4). In the infant’s gut microbiota, LEfSe detected one bacterial family—Enterobacteriaceae, belonging to the phylum Proteobacteria (class Gammaproteobacteria, order Enterobacteriales) which was discriminating the threonine low and high group. After adjustment for FDR (using 85 taxa), significant differences remained for Proteobacteria (*p* = 0.028) and Gammaproteobacteria (*p* = 0.028), but only a trend for Enterobacteriales/Enterobacteriaceae (*p* = 0.077).

Taking advantage of the possibility to include one important confounder, LEfSe was also performed using the established confounder “birth mode” in the subclass analysis. As described above, LEfSe detected Enterobacteriaceae, discriminating the threonine low and high group (Fig. 4a), irrespective of birth mode. Data are presented as log10-transformed LDA scores (where LDA scores of ≥ 2.0 were considered relevant (with *p*< 0.05)) and in the form of a cladogram (Fig. 4b). The distribution of Enterobacteriaceae by threonine class and birth mode subclass is shown in Fig. 4c.

### Multivariable regression models

To confirm the above results and to adjust for potential confounders, the bacterial groups resulting from LEfSe were analyzed together with the continuous amino acid values in univariate and multivariate linear regression models. Therefore, threonine first was subjected to univariate regression analyses with the relative abundance table of taxonomic groups on all levels. The results indicated that the following bacterial groups exhibited significant association with threonine: Proteobacteria, Gammaproteobacteria, Enterobacteriales, Enterobacteriaceae, and genera Escherichia/Shigella. In order to account for potential confounders all regression models were adjusted for the literature based confounders GWG, feeding mode, mode of birth, infant sex and parity (unless stated otherwise).

The results of the regression models (adjusted for confounders) are presented in Tables 5, 6. Notably, regression analysis revealed a strong negative association of threonine with the genus Escherichia/Shigella (family Enterobacteriaceae, class Gammaproteobacteria, phylum Proteobacteria) (Table 5). When the models were adjusted with a more stringent feeding type classification (exclusively breast-fed infants (*n* = 31 versus rest *n* = 10) instead of predominantly breast-fed infants (*n* = 35 versus mixed *n* = 6)), the association of threonine with the identified bacterial entities was even slightly improved (Table 5 and full model in online resource 5). In order to demonstrate the magnitude of the **additive predictive effect of human milk free threonine** on the abundance of Enterobacteriales beyond confounders, the contribution of all variables is shown in Table 6. As indicated in Table 6, the relative threonine concentration in breast milk had the strongest predictive effect (predicting the abundance of Enterobacteriales) when compared to the effects of the confounders.

**Table 5:** Linear regression analysis of human milk free threonine (in %) with bacterial entities adjusted for confounders. Predominantly breastfed (BF) infants and exclusively BF infants (*) ^a^

Regression models	*β*	Lower bound 95% CI	Upper bound 95% CI	*P* value	*R*^2^ (%)	adj.R^**2**^ (%)	β*	p*	adj. R^**2**^(%)*	*n*
Proteobacteria (Phyla)										
Constant		0.522	3.144	0.007						
Threonine LOG	− 0.441	− 7.368	− 1.308	**0.006**	**19.4**	**17.1**	− 0.442	**0.006**	**17.3**	37
Constant		− 0.092	3.069	0.064						
Alanine	− 0.313	− 0.436	0.009	0.060	9.8	7.2	− 0.332	**0.045**	**8.5**	37
Gammaproteobacteria (Class)										
Constant		0.752	3.303	0.003						
Threonine LOG	− 0.488	− 7.745	− 1.851	**0.002**	**23.8**	**21.6**	− 0.484	**0.002**	**21.2**	37
Constant		− 0.064	3.092	0.059						
Alanine	− 0.318	− 0.439	0.005	0.055	10.1	7.5	− 0.326	**0.049**	**8.1**	37
Enterobacteriales/Enterbacteriaceae (Order/Fam)										
Constant		0.831	3.355	0.002						
Threonine LOG	− 0.504	− 7.871	− 2.037	**0.001**	**25.4**	**23.2**	− 0.515	**0.001**	**24.4**	37
Escherichia_Shigella (Genus)										
Constant		0.172	2.888	0.028						
Threonine LOG	− 0.368	− 6.760	− 0.482	**0.025**	**13.6**	**11.1**	− 0.392	**0.016**	**12.9**	37

Predicting amino acids were determined breast milk; only significant results are shown (*p* < 0.05)

Beta = standardized regression coeffient, CI = Confidence Interval, R^2^ = coefficient of determination, adj R^2^ = adjusted coefficient of determination, *n*= numbers of included participants (n is reduced to 37 as not all the parameters including confounders could be determined in every mother infant pair); confounders: GWG, parity, feeding mode, birth mode, infant sex

**Table 6:** Complete model: linear regression analysis of human milk free threonine (in %) with Enterobacteriales showing the additive effect of threonine on the predictive effect of common confounders

Regression models	*β*	Lower bound 95% CI	Upper bound 95% CI	*p* value	*R*^2^ (%)	adj.* R*^2^ (%)	*n*
Enterobacteriales complete model					40.8	28.9	37
(Constant)		0.253	0.885	0.001			
**Threonine **LOG	**− 0.54**	− 0.957	− 0.258	**0.001**			
Infant Sex	0.227	− 0.018	0.113	0.151			
GWG LOG	− 0.156	− 0.388	0.118	0.284			
Birth Mode	0.148	− 0.035	0.099	0.339			
Feeding mode	0.038	− 0.082	0.105	0.802			
Parity	0.041	− 0.070	0.090	0.799			

Significant impacts are highlighted in bold

*GWG* gestational weight gain

Beta = standardized regression coeffient, CI = Confidence Interval, R^2^ = coefficient of determination, adj R^2^ = adjusted coefficient of determination, *n*= numbers of included participants (n is reduced to 37 as not all the parameters including confounders could be determined in every mother infant pair); confounders: GWG, parity, feeding mode, birth mode, infant sex

Therefore, our final result shows that threonine appears to be significantly negatively associated with the abundance of members of the phylum Proteobacteria.

## Discussion

In general, dietary amino acids are thought to be important players in gut physiology and health, and recent research suggests that the microbiota is a considerable/effective component of this system (Beaumont and Blachier 2020). In infants, the effect of AA might be pivotal with regard to maturation of the immune system and with regard to nutritional programming. In breast milk-fed infants (before the introduction of solid foods; as in our analysis time point at 2 months), the amino acid composition of breast milk determines the amount of amino acids the infants gut (and gut microbiota) get in contact with. Many studies already focused on the effect of the total protein concentration in infant nutrition (especially in formula-fed infants), which is thought to increase the long-term risk for obesity in childhood (Koletzko et al. 2019). However, the importance of bioactive FAA has been less exploited so far. Only few data are available that address a possible relationship between maternal serum and breast milk FAA (Ramírez et al. 2001), providing results that may indicate underlying mechanisms for the emergence of these different FAA patterns in breast milk. Furthermore, hardly any study addressed the FAA composition of breast milk in lactation in relation to the infants’ intestinal microbiota (He et al. 2020; Kok et al. 2020).

In our study cohort, seven FAA we found to be highly correlated between maternal serum and breast milk samples. Interestingly, all of them belong to the group of FAA that are lower in human milk than in serum (ratio = 0.17–0.83). In an attempt to fit these results into the existing theories on the origin of breast milk FAA profiles, it could be assumed that these FAA in milk emerged due to a directed (F)AA transport from blood into the breast milk. As FAA concentration was shown to reflect maternal dietary protein intake in early studies (Robert G. Jensen, 1995; Newburg et al. 2001), the optimal dietary supply of the lactating mothers with balanced amino acids comes back into focus, eventually or especially for those obviously connected FAA species.

Concerning maternal diet and its influence of infant microbiota, so far, only high-fat diet, vitamins and fibers have been linked to infant microbiota composition (Chu et al. 2017; Maher et al. 2020). With our results we provide the first evidence that one of these highly correlated FAA, threonine from breast milk, is also associated with the composition of the infant microbiota. The general microbiota composition in our cohort is in accordance with other studies (Cukrowska et al. 2020; Solís et al. 2010) investigating predominantly breast-fed infants at a similar time point of lactation, with Bifidobacteria as the most predominant family. Adonis association analyses revealed a significant association of beta diversity with the FAA threonine in breast milk (with 7% of the variation in distances being explained by the FAA threonine).

When beta diversity was addressed into more detail using LEfSe effect size calculations (taking into account biological effect size), several microbial entities were proposed to be significantly associated with threonine (categorized in high/low classes). With multivariate regression analyses we were able to confirm some of the proposed associations, which even remained significant after adjustment for literature based confounders: In our study cohort of healthy mother infant dyads, we identified a lower relative abundance of Proteobacteria, Gammaproteobacteria, Enterobacteriaceae and its genus Escherichia/Shigella in infants fed with breast milk containing higher amounts of FAA threonine. This might be indicative of a potential protective role of threonine against members of the Enterobacteriaceae as e.g. Escherichia/Shigella, which two clades cannot be separated well on the basis of the 16S rRNA gene sequence.

The phylum Proteobacteria is believed to be an important contributor to inflammation associated with metabolic disease in adults and they might also play a role in the priming of the infants immune system (Mulligan and Friedman 2017). Furthermore, Proteobacteria were described to explain significant functional variability of the human intestinal microbiota—as they emerged as a major source of variable genes (Bradley and Pollard 2017). The class of Gammaproteobacteria e.g. has already been brought into context with maternal factors, as neonates born to mothers with obesity showed a 50% reduction in Gammaproteobacteria at 2 weeks of age compared with infants of normal-weight mothers (Lemas et al. 2016).

The family of Enterobacteriaceae is described to be the second or third largest family in infant fecal microbiota (Ma et al. 2020; Fallani et al. 2010), which is in line with the results of our study. However, they are quite undesirable in neonates, as members of the family Enterobacteriaceae are involved in nosocomial infections and increased abundance of Enterobacteriaceae determines a risk factor for necrotizing enterocolitis (NEC) and sepsis, especially in the preterm population (Neu and Pammi 2017; Greenwood et al. 2014).

Threonine (also known as α-amino-β-hydroxybutyric acid) is an essential amino acid that has two metabolic fates in the intestine: (a) incorporation into mucosal proteins including mucins and (b) catabolism by luminal commensales (Stoll et al. 1998). Threonine supplementation or dietary threonine concentration has already been brought into context with improved gut integrity and function in cell and tissue culture studies as well as in animal models (Koo et al. 2020; Feng et al. 2013). In those studies, threonine supplementation increased villus height and goblet cell density as well as tight junction protein (occludin and zonulin) gene expression (Hamard et al. 2010) and enhanced mucus production (Trevisi et al. 2015; Law et al. 2007; Chen et al. 2017). Furthermore, dietary threonine restriction is described to decrease the production of digestive enzymes that are abundant in threonine and to negatively affect intestinal barrier (Block et al. 1966., n.d.; Hamard et al. 2010).

In consent with our results of reduced Enterobacteriaceae with high threonine levels, Trevisi et al. (Trevisi et al. 2015) reported that diet supplemented with 9.0 g threonine/kg reduced fecal *E. coli* counts in pigs experimentally challenged with *E.coli*. Furthermore, they showed that threonine—as important component of immunoglobulins—significantly induced IgG and IgA antibody formation (Trevisi et al. 2015), indicating its involvement in (immuno-) protective pathways. This immunological context is also substantiated by the fact that in conditions of inflammation, infection, disease (colonic carcinoma, HIV, sepsis,..) or other immunological challenges, the threonine requirement of the intestinal mucosa was increased (Dawson and Filipe 1982; Laurichesse et al. 1998; Mao et al. 2011).This all together implicates the potential impact of dietary threonine on gut (barrier) integrity, host protection and immune function, essential processes in the maturation of the infants’ immune system.

Antibiotics:

Interestingly, the treatment of mother and/or infant with antibiotics (including intrapartum antimicrobial prophylaxis) was also shown to increase the abundance of Enterobacteriaceae (Greenwood et al. 2014) in the infant, as they harbor more resistance genes than many other gut clades. To address this point, we also included intrapartum antibiotics in an additional model with threonine, but did not observe a different result as compared to the model without antibiotics (data not shown, Enterobacteriaceae: R2 adj = 22.8%, *p* = 0.002; beta = − 0.501; Escherichia/Shigella: R2 adj = 9.8%; *p* = 0.038; beta = − 0.353). Interestingly, in pigs, treatment with antibiotics resulted in elevated threonine plasma concentrations and turnover (Puiman et al. 2013).

Food allergy:

Several diseases such as food allergy and obesity have also been associated with increased Enterobacteriaceae especially with the increased ratio of Enterobacteriaceae to Bacteroidaceae (Dong et al. 2018; Wu 2010; Azad et al. 2015). In our cohort, however, milk threonine did not have any influence on this aforementioned ratio (data not shown). Preterm infants also show an altered gut microbiota composition, with increased abundance of Enterobacteriaceae (Mai et al. 2011), but in our cohort, we were only investigating term-delivered infants.

Infant formula:

Formula-fed infants are a heterogenous group, also in terms of FAA supply. In the majority of conventional infant formulas, threonine is predominantly present in its protein-bound form and only in smaller amounts in its free form (Agostoni et al. 2000). In a more recent study performed by Ventura et al. free threonine was even detected only in modified formulas in which proteins are hydrolyzed, as in partially or extensively hydrolyzed formulations that were developed to reduce the risk of allergy (Alison K. Ventura et al. 2012a, b). This altogether—and especially the huge differences in FAA content between the different formula types (Ventura et al.)—rather excludes formula-fed infants from further comparisons or considerations towards the amount of Enterobacteriaceae (at least without further specification of the formula group).

Nevertheless, the threonine metabolism seems to be interesting, as compared to infants fed breast milk, standard formula-fed infants exhibited higher plasma threonine concentrations that could not be explained by differences in threonine intake (Darling et al. 1999). Therefore, formula-fed infants were thought to possess a lower capacity to oxidize and degrade threonine (in response to higher threonine loads) than breast milk-fed infants (Darling et al. 1999), leading to different threonine levels in serum. The underlying mechanisms have not been resolved yet, but this could be due to the altered composition of intestinal bacterial communities found in formula-fed infants with different capability to oxidize threonine. Stool samples of formula-fed infants e.g. exhibit increased alpha diversity and reduced amounts of Bifidobacteria in the first months of life (Ma et al. 2020), probably due to the lack of exposure to beneficial components of breast milk, including the milk microbiota.

In our study cohort, however, the majority of infants were predominantly breastfed (*n* = 35). No infants were exclusively formula fed and only six were mixed feeders, so this effect is not relevant for our cohort. This was also reflected by the fact that the adjustment for feeding mode in the regression models did not affect the model strength. In order to exclude a possible influence by small amounts of formula in the predominantly breast-fed group, the variable feeding mode was reclassified as “exclusively breast fed” (= non formula at all, from birth to 2 months, *n* = 31) versus “other” (*n* = 10). This reclassification did not change the regression models of threonine significantly, except that the models were slightly improved.

Decreased intestinal threonine metabolism and subsequently impaired gut barrier function were suspected to predispose the formula-fed infant to developing NEC in pigs (Puiman et al. 2013). But as obvious from our study cohort, dietary threonine might also have an influence on the microbiota composition of healthy term breast-fed infants.

It would be essential to understand the mechanisms by which the dietary FAA profile may influence the intestinal microbiota of infants. Undoubtedly, many AA contribute to the survival and proliferation of the microbiota after being metabolized by microorganisms. An additional mechanism (for AA to impact microbiota composition) might be the upregulation of beta-defensin (an antimicrobial peptide, produced by intestinal cells), which was nicely demonstrated in AA-supplemented piglets (Yi et al. 2018).

Microbes do not only consume AA but also can provide (essential) AA and other metabolites as e.g. short chain fatty acids (SCFA) to the host. Propionate and butyrate, are the two SCFA considered to beneficially effect health, by supplying energy to the gut mucosa in the case of butyrate, and by induction of gluconeogenesis and promotion of satiety in the case of propionate (Morrison and Preston, 2016). Interestingly, our identified amino acid threonine is one of the major progenitors of propionate (Louis and Flint, 2017), although the numbers of AA-fermenting bacteria is quite low in the large intestine.

Due to the pilot nature of the study (*n* = 41), results have to be considered with caution and have to be confirmed in a larger study cohort. Furthermore, the results are based on correlation analyses and have to be confirmed and strengthened by functional studies to provide information about potential causality. A limitation of the study might result from the fact that serum AA analysis was carried out in non-fasting blood samples of lactating mothers. Additionally, other components of breast milk (as human milk oligosaccharides, milk microbiota) might also be relevant for the development on the infant intestinal microbiota composition. However, our study cohort was very homogenous and well characterized, thereby strengthening our results.

Finally, our study suggests potential interactions between breast milk FAA and infant gut microbiota that in turn might have the potential to effect gut development, immunological and metabolic health and might be a component of nutritional programming of the infant. We here provide evidence that threonine might be especially relevant in the early lactational period. Therefore, a detailed elucidation of maternal dietary AA supply in lactation together with infant microbiota composition might be worth further studies. Our results show that breast milk FAA are associated with infant intestinal microbiota composition and they are pointing towards a previously unidentified strong negative association of the breast milk FAA threonine with members of the phylum Proteobacteria. Further studies with larger cohorts are needed to support the findings and elucidate possible mechanisms in order to assess the complex interplay between dietary milk FAA and infant intestinal microbiota into detail.

## Supplementary Information

Below is the link to the electronic supplementary material.

Supplementary file1 (PDF 2283 kb)

## Data Availability

The datasets generated and/or analyzed during the current study are not publicly available due to data security issues (the informed consent did not include this option) but are available from the corresponding author on reasonable request.

## References

[CR1] Agostoni C (2000). Free Amino Acid Content in Standard Infant Formulas: Comparison with Human Milk. J Am Coll Nutr.

[CR2] Alemán G (2009). Changes in messenger RNA abundance of amino acid transporters in rat mammary gland during pregnancy, lactation, and weaning. Metabolism.

[CR3] Anderson MJ (2001). A new method for non-parametric multivariate analysis of variance. Austral Ecol.

[CR4] Anon (n.d.) *WHO | WHO Anthro (version 3.2.2, January 2011) and macros* [online]. Available from: http://www.who.int/childgrowth/software/en/. Accessed 18 April 2019.

[CR5] Anon (n.d.) *LEfSe* [online]. Available from: https://galaxyproject.org/learn/visualization/custom/lefse/. Accessed 9 March 2020.

[CR6] Anon (2021) *adonis function | R Documentation [Internet].*

[CR7] Azad MB (2015). Infant gut microbiota and food sensitization: associations in the first year of life. Clin Exp Allergy.

[CR8] Baldeón (2019). Free Amino Acid Content in Human Milk is Associated with Infant Gender and Weight Gain during the First Four Months of Lactation. Nutrients.

[CR9] Beaumont M, Blachier F, Guoyao Wu (2020). Amino Acids in Intestinal Physiology and Health. Amino acids in nutrition and health advances in experimental medicine and biology.

[CR10] Block RJ, Weiss KW, Cornett DB, Composition TAA, of Proteins and Foods. R. J. Block and D. Bolling, (1966). (n.d.) ‘*The amino acid composition of proteins*. Charles C.

[CR11] Bolyen E (2019). Reproducible, interactive, scalable and extensible microbiome data science using QIIME 2. Nat Biotechnol.

[CR12] Bradley PH, Pollard KS (2017). Proteobacteria explain significant functional variability in the human gut microbiome. Microbiome.

[CR13] Callahan BJ (2016). DADA2: high-resolution sample inference from illumina amplicon data. Nat Methods.

[CR14] Carratù B (2003). Nitrogenous components of human milk: non-protein nitrogen, true protein and free amino acids. Food Chem.

[CR15] Chen YP (2017). Effects of threonine supplementation on the growth performance, immunity, oxidative status, intestinal integrity, and barrier function of broilers at the early age. Poult Sci.

[CR16] Chu DM (2017). Maturation of the infant microbiome community structure and function across multiple body sites and in relation to mode of delivery. Nat Med.

[CR17] Cox LM (2014). Altering the Intestinal Microbiota during a Critical Developmental Window Has Lasting Metabolic Consequences. Cell.

[CR18] Cukrowska B (2020). The Relationship between the Infant Gut Microbiota and Allergy. The Role of Bifidobacterium breve and Prebiotic Oligosaccharides in the Activation of Anti-Allergic Mechanisms in Early Life. Nutrients.

[CR20] Dallas DC (2012) Digestion of Protein in Premature and Term Infants. *Journal of Nutritional Disorders & Therapy*. 02:03. https://www.omicsonline.org/digestion-of-protein-in-premature-and-term-infants-2161-0509.1000112.php?aid=6036. Accessed 29 March 2021. 10.4172/2161-0509.1000112 PMC3988022 24744976

[CR19] Dallas DC (2015). Proteolytic Systems in Milk: Perspectives on the Evolutionary Function within the Mammary Gland and the Infant. J Mammary Gland Biol Neoplasia.

[CR21] Darling PB (1999). Threonine kinetics in preterm infants fed their mothers’ milk or formula with various ratios of whey to casein. Am J Clin Nutr.

[CR22] Dawson PA, Filipe MI (1982). Uptake of [3H]threonine in human colonic mucosa associated with carcinoma: An autoradiographic analysis at the ultrastructural level. Histochem J.

[CR23] Dong P (2018). Early-life gut microbiome and cow’s milk allergy- a prospective case - control 6-month follow-up study. Saudi Journal of Biological Sciences.

[CR24] Faith DP (1992). Conservation evaluation and phylogenetic diversity. Biol Cons.

[CR25] Fallani M (2010). Intestinal microbiota of 6-week-old infants across europe: geographic influence beyond delivery mode, breast-feeding, and Antibiotics. J Pediatr Gastroenterol Nutr.

[CR26] Feng L (2013). Threonine affects intestinal function, protein synthesis and gene expression of TOR in Jian Carp (Cyprinus carpio var. Jian) Daniel Merrifield (ed.). PLoS ONE.

[CR27] Field CJ (1994). Enhanced metabolism of glucose and glutamine in mesenteric lymph node lymphocytes from spontaneously diabetic BB rats. Can J Physiol Pharmacol.

[CR28] Garcia-Rodenas C (2016). Amino acid composition of breast milk from urban chinese mothers. Nutrients.

[CR29] Greenwood C (2014). Early empiric antibiotic use in preterm infants is associated with lower bacterial diversity and higher relative abundance of enterobacter. J Pediatr.

[CR30] Hamard A (2010). A moderate threonine deficiency affects gene expression profile, paracellular permeability and glucose absorption capacity in the ileum of piglets. J Nutr Biochem.

[CR31] He X (2020). The role of protein and free amino acids on intake, metabolism, and gut microbiome: a comparison between breast-fed and formula-fed rhesus monkey infants. Front Pediatr.

[CR32] Robert G. Jensen (1995) *Handbook of Milk Composition*. [Online]. Elsevier. https://linkinghub.elsevier.com/retrieve/pii/B9780123844309X50008. Accessed 2 April 2021.

[CR33] Jochum F (2006). Total glutamine content in human milk is not influenced by gestational age. Acta Paediatr.

[CR34] Katoh K, Standley DM (2013). MAFFT multiple sequence alignment software version 7: improvements in performance and usability. Mol Biol Evol.

[CR35] Keylock CJ (2005). Simpson diversity and the Shannon-Wiener index as special cases of a generalized entropy. Oikos.

[CR36] Kok CR (2020). Stool microbiome, pH and short/branched chain fatty acids in infants receiving extensively hydrolyzed formula, amino acid formula, or human milk through two months of age. BMC Microbiol.

[CR37] Koletzko B (2019). Optimized protein intakes in term infants support physiological growth and promote long-term health. Semin Perinatol.

[CR38] Koo B (2020). Diet complexity and l-threonine supplementation: effects on growth performance, immune response, intestinal barrier function, and microbial metabolites in nursery pigs. J Anim Sci.

[CR39] Koopman R (2009). Ingestion of a protein hydrolysate is accompanied by an accelerated in vivo digestion and absorption rate when compared with its intact protein. Am J Clin Nutr.

[CR40] Labbok M, Krasovec K (1990). Toward consistency in breastfeeding definitions. Stud Fam Plann.

[CR41] Larnkjær A (2016). Free amino acids in human milk and associations with maternal anthropometry and infant growth. J Pediatr Gastroenterol Nutr.

[CR42] Laurichesse H (1998). Threonine and methionine are limiting amino acids for protein synthesis in patients with AIDS. J Nutr.

[CR43] Law GK (2007). Adequate oral threonine is critical for mucin production and gut function in neonatal piglets. Am J Physiol Gastrointest Liver Physiol.

[CR44] Lemas DJ (2016). Alterations in human milk leptin and insulin are associated with early changes in the infant intestinal microbiome. Am J Clin Nutr.

[CR45] Lin Y (2018). The effects of L-type amino acid transporter 1 on milk protein synthesis in mammary glands of dairy cows. J Dairy Sci.

[CR46] Louis P, Flint HJ (2017). Formation of propionate and butyrate by the human colonic microbiota. Environ Microbiol.

[CR47] Lozupone CA (2007). Quantitative and qualitative β diversity measures lead to different insights into factors that structure microbial communities. Appl Environ Microbiol.

[CR48] Lozupone C, Knight R (2005). UniFrac: a new phylogenetic method for comparing microbial communities. Appl Environ Microbiol.

[CR49] Ma J (2020). Comparison of gut microbiota in exclusively breast-fed and formula-fed babies: a study of 91 term infants. Sci Rep.

[CR50] Maher SE (2020). The association between the maternal diet and the maternal and infant gut microbiome: a systematic review. Br J Nutr.

[CR51] Mai V (2011). Fecal microbiota in premature infants prior to necrotizing enterocolitis Dipshikha Chakravortty (ed.). PLoS ONE.

[CR52] Mao X (2011). Specific roles of threonine in intestinal mucosal integrity and barrier function. Front Biosci.

[CR53] Morrison DJ, Preston T (2016). Formation of short chain fatty acids by the gut microbiota and their impact on human metabolism. Gut Microbes.

[CR54] Mulligan CM, Friedman JE (2017). Maternal modifiers of the infant gut microbiota: metabolic consequences. J Endocrinol.

[CR55] Neu J, Pammi M (2017). Pathogenesis of NEC: Impact of an altered intestinal microbiome. Semin Perinatol.

[CR56] Newburg, D. S. et al. (2001) *Bioactive Components of Human Milk*. http://proxy.lib.utk.edu:90/login?url=http://dx.doi.org/10.1007/978-1-4615-1371-1. Accessed 2 April 2021.

[CR57] Price MN (2010). FastTree 2 approximately maximum-likelihood trees for large alignments art F. Y. Poon (ed.). PLoS ONE.

[CR58] Puiman P (2013). Modulation of the gut microbiota with antibiotic treatment suppresses whole body urea production in neonatal pigs. Am J Physiol Gastrointest Liver Physiol.

[CR59] Quast C (2012). The SILVA ribosomal RNA gene database project: improved data processing and web-based tools. Nucleic Acids Res.

[CR60] Ramírez I, Newburg David S (2001). Amino Acid Intake During Lactation and Amino Acids of Plasma and Human Milk. Bioactive components of human milk. Advances in experimental medicine and biology.

[CR61] Riederer M (2020). Distinct maternal amino acids and oxylipins predict infant fat mass and fat-free mass indices. Arch Physiol Biochem.

[CR62] Roth E (2007). Immune and cell modulation by amino acids. Clin Nutr.

[CR63] Ruth MR, Field CJ (2013). The immune modifying effects of amino acids on gut-associated lymphoid tissue. J Anim Sci Biotechnol.

[CR64] van Sadelhoff J (2018). Longitudinal Variation of Amino Acid Levels in Human Milk and Their Associations with Infant Gender. Nutrients.

[CR65] van Sadelhoff JHJ (2020). Free amino acids in human milk: a potential role for glutamine and glutamate in the protection against neonatal allergies and infections. Front Immunol.

[CR66] Schanler RJ, Garza C (1987). Plasma amino acid differences in very low birth weight infants fed either human milk or whey-dominant cow milk formula. Pediatr Res.

[CR67] Segata N (2011). Metagenomic biomarker discovery and explanation. Genome Biol.

[CR68] Solís G (2010). Establishment and development of lactic acid bacteria and bifidobacteria microbiota in breast-milk and the infant gut. Anaerobe.

[CR69] Stoll B (1998). Catabolism dominates the first-pass intestinal metabolism of dietary essential amino acids in milk protein-fed piglets. J Nutr.

[CR70] Trevisi P (2015). Effect of added dietary threonine on growth performance, health, immunity and gastrointestinal function of weaning pigs with differing genetic susceptibility to *Escherichia coli* infection and challenged with *E. coli* K88ac. J Anim Physiol Anim Nutr.

[CR71] Vandenplas, Y. et al. (2020) Factors affecting early-life intestinal microbiota development. *Nutrition*. [Online] 78110812. 10.1016/j.nut.2020.110812 32464473

[CR72] Vázquez-Baeza Y (2013). EMPeror: a tool for visualizing high-throughput microbial community data. GigaScience.

[CR73] Ventura AK (2012). Free amino acid content in infant formulas. Nutr Food Sci.

[CR74] Ventura Alison K (2012). Infant regulation of intake: the effect of free glutamate content in infant formulas. Am J Clin Nutr.

[CR75] Wells JCK (2014). Toward body composition reference data for infants, children, and adolescents. Adv Nutr.

[CR76] World Health Organization (2008) *Indicators for assessing infant and young child feeding practices: part 1: definitions: conclusions of a consensus meeting held 6–8 November 2007 in Washington D.C., USA*. https://apps.who.int/iris/handle/10665/43895. Accessed 12 August 2020.

[CR77] Wu G (2010). Functional amino acids in growth, reproduction, and health. Adv Nutr.

[CR78] Wu G (2013). Functional amino acids in nutrition and health. Amino Acids.

[CR79] Yamawaki N (2005). Macronutrient, mineral and trace element composition of breast milk from Japanese women. J Trace Elem Med Biol.

[CR80] Yi D (2018). Dietary supplementation with an amino acid blend enhances intestinal function in piglets. Amino Acids.

[CR81] Zhang Z (2013). Amino acid profiles in term and preterm human milk through lactation: a systematic review. Nutrients.

[CR82] Ziegler TR (2003). Trophic and cytoprotective nutrition for intestinal adaptation, mucosal repair, and barrier function. Ann Rev Nutr.

