# Supplementary material for: Free threonine in human breast milk is related to infant intestinal microbiota composition
Source: Amino Acids. 2021 Sep 3;54(3):365–83. doi: 10.1007/s00726-021-03057-w (PMC8948153; doi:10.1007/s00726-021-03057-w)
Supplement: Supplementary file 1 — Supplementary file1 (PDF 2283 kb) [file 726_2021_3057_MOESM1_ESM.pdf]

Online Resources:

Free threonine in human breast milk is related to infant intestinal microbiota composition

Riederer Monika, Schweighofer Natascha, Trajanoski Slave, Stelzer Claudia, Zehentner Miriam, Fuchs-Neuhold Bianca, Kashofer Karl, Johannes A. Mayr, Hörmann-Wallner Marlies, Holasek Sandra, Moenie van der Kleyn*

Online resource 1: Maternal baseline characteristics for FAA comparison in serum and breastmilk (n=41). This cohort was used for comparison of FAA in maternal serum and breast milk.

Online resource 2 Correlation Matrix

a) Correlation matrix of distinct normally distributed serum and human milk (HM) FAA. Colors and values indicate Pearson correlation coefficients.

b) Correlation matrix of distinct not normally distributed serum and HM FAA. Colors and values indicate Spearman correlation coefficients.

Online resource 3 Infant intestinal microbiota composition, presented as relative abundance

a) Table of the most abundant taxa (with more than 10%)
b) Table of all taxa

Online resource 4 a-c Alpha-diversity indices

Alpha-diversity measures richness (a), Shannon (b), Faiths phylogenetic diversity (PD) (c) were analyzed in threonine classes (high and low; threshold: 2.69%). Differences between classes were analyzed using Wilcoxon tests. Only richness was significantly increased in the high threonine group (p=0.03188).

Online resource 5: Linear regression analysis of human milk free threonine (in %) with bacterial entities adjusted for confounders (a) versus entities adjusted for confounders including exclusively breast fed category (b) [c]

[c]Predicting amino acids were determined breast milk ; only significant results are shown ($p< 0.05$), beta = standardized regression coeffient, CI=Confidence Interval, R^2=coefficient of determination, adj R2 = adjusted coefficient of determination, n=numbers of included participants (n is reduced as not all the parameters including confounders could be determined in every mother infant pair); confounders (a): GWG, parity, feeding mode (predominantly breast fed versus rest), birth mode, infant sex; confounders (b): GWG, parity, feeding mode (exclusively breast fed versus rest), birth mode, infant sex;

Online resource 1: Maternal baseline characteristics for FAA comparison in serum and breastmilk (n=41) [a]

Maternal characteristics	**% or mean**	**SD**	**n**
austrian nationality	78.0		32
Maternal age at delivery (years)	31.7	3.6	41
Early pregnancy BMI (kg/m²)	22.6	2.9	38
Early pregnancy BMI category			
Low (BMI ≥ 18,5 to < 25 kg/m²)	82.9		34
High (BMI ≥ 25 to ≤30.5 kg/m²)	17.1		7
Gestational weight gain (kg)	14.5		38
Parity (%)			
primipara	75.6		31
multipara	24.4		10
Caesarean delivery (%)	36.6		15
Antibiotics use pre- or during birth (%)			
no	69.2		27
yes	30.8		12
Breastfeeding practices at 2 mths (%)			
exclusive breastfeeding	80.5		33
breastfeeding plus < 30 ml formula	7.3		3
predominantly breastfed	87.8		36
mixed fed	12.2		5

online resource 2a

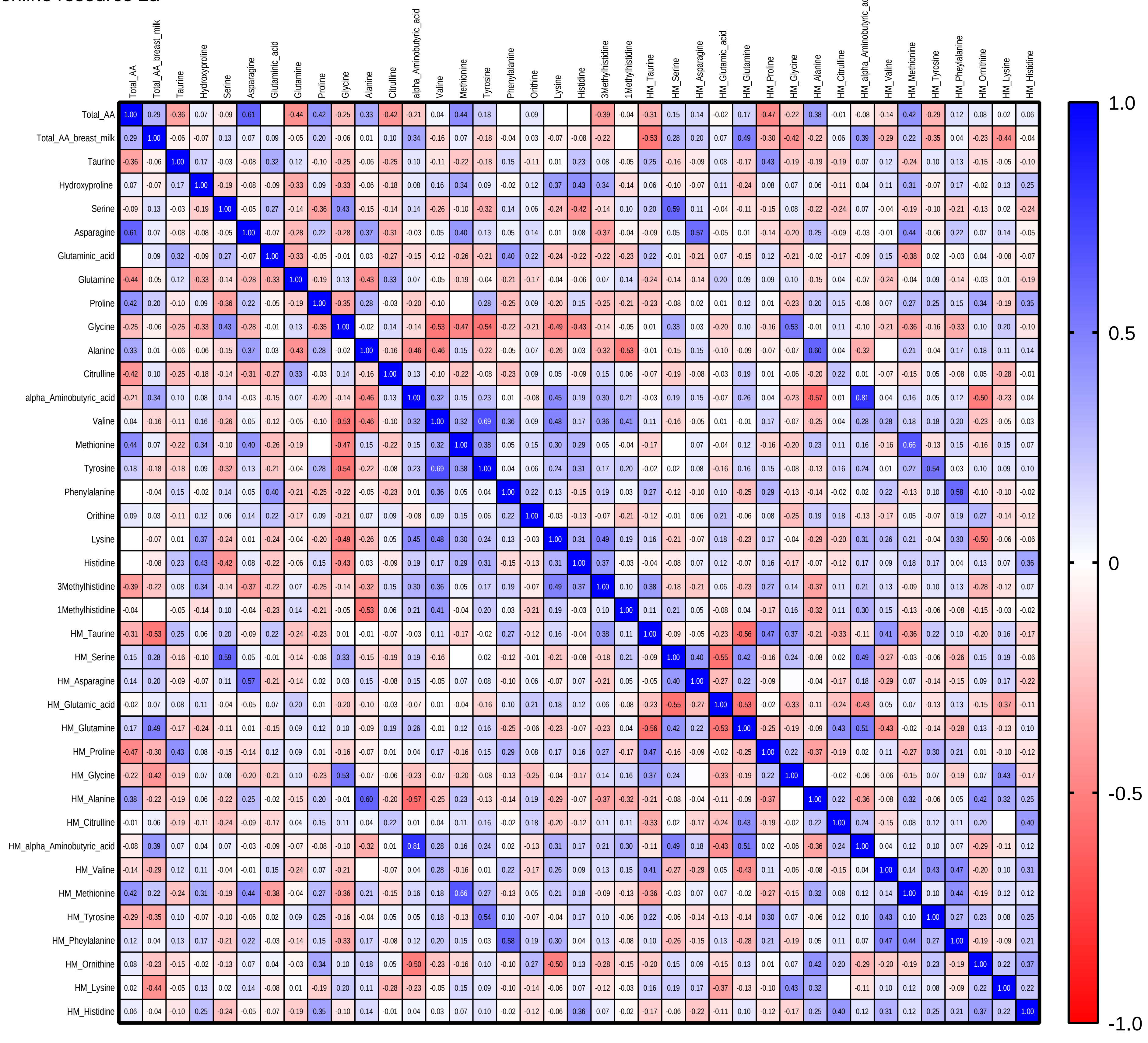

	Total_AA	Total_AA_breast_milk	Taurine	Hydroxyproline	Serine	Asparagine	Glutaminic_acid	Glutamine	Proline	Glycine	Alanine	Citrulline	alpha_Aminobutyric_acid	Valine	Methionine	Tyrosine	Phenylalanine	Orithine	Lysine	Histidine	3Methylhistidine	1Methylhistidine	HM_Taurine	HM_Serine	HM_Asparagine	HM_Glutamic_acid	HM_Glutamine	HM_Proline	HM_Glycine	HM_Alanine	HM_Citrulline	HM_alpha_Aminobutyric_acid	HM_Valine	HM_Methionine	HM_Tyrosine	HM_Pheylalanine	HM_Ornithine	HM_Lysine	HM_Histidine
Total_AA	1.00	0.29	-0.36	0.07	-0.09	0.61		-0.44	0.42	-0.25	0.33	-0.42	-0.21	0.04	0.44	0.18		0.09			-0.39	-0.04	-0.31	0.15	0.14	-0.02	0.17	-0.47	-0.22	0.38	-0.01	-0.08	-0.14	0.42	-0.29	0.12	0.08	0.02	0.06
Total_AA_breast_milk	0.29	1.00	-0.06	-0.07	0.13	0.07	0.09	-0.05	0.20	-0.06	0.01	0.10	0.34	-0.16	0.07	-0.18	-0.04	0.03	-0.07	-0.08	-0.22		-0.53	0.28	0.20	0.07	0.49	-0.30	-0.42	-0.22	0.06	0.39	-0.29	0.22	-0.35	0.04	-0.23	-0.44	-0.04
Taurine	-0.36	-0.06	1.00	0.17	-0.03	-0.08	0.32	0.12	-0.10	-0.25	-0.06	-0.25	0.10	-0.11	-0.22	-0.18	0.15	-0.11	0.01	0.23	0.08	-0.05	0.25	-0.16	-0.09	0.08	-0.17	0.43	-0.19	-0.19	-0.19	0.07	0.12	-0.24	0.10	0.13	-0.15	-0.05	-0.10
Hydroxyproline	0.07	-0.07	0.17	1.00	-0.19	-0.08	-0.09	-0.33	0.09	-0.33	-0.06	-0.18	0.08	0.16	0.34	0.09	-0.02	0.12	0.37	0.43	0.34	-0.14	0.06	-0.10	-0.07	0.11	-0.24	0.08	0.07	0.06	-0.11	0.04	0.11	0.31	-0.07	0.17	-0.02	0.13	0.25
Serine	-0.09	0.13	-0.03	-0.19	1.00	-0.05	0.27	-0.14	-0.36	0.43	-0.15	-0.14	0.14	-0.26	-0.10	-0.32	0.14	0.06	-0.24	-0.42	-0.14	0.10	0.20	0.59	0.11	-0.04	-0.11	-0.15	0.08	-0.22	-0.24	0.07	-0.04	-0.19	-0.10	-0.21	-0.13	0.02	-0.24
Asparagine	0.61	0.07	-0.08	-0.08	-0.05	1.00	-0.07	-0.28	0.22	-0.28	0.37	-0.31	-0.03	0.05	0.40	0.13	0.05	0.14	0.01	0.08	-0.37	-0.04	-0.09	0.05	0.57	-0.05	0.01	-0.14	-0.20	0.25	-0.09	-0.03	-0.01	0.44	-0.06	0.22	0.07	0.14	-0.05
Glutaminic_acid		0.09	0.32	-0.09	0.27	-0.07	1.00	-0.33	-0.05	-0.01	0.03	-0.27	-0.15	-0.12	-0.26	-0.21	0.40	0.22	-0.24	-0.22	-0.22	-0.23	0.22	-0.01	-0.21	0.07	-0.15	0.12	-0.21	-0.02	-0.17	-0.09	0.15	-0.38	0.02	-0.03	0.04	-0.08	-0.07
Glutamine	-0.44	-0.05	0.12	-0.33	-0.14	-0.28	-0.33	1.00	-0.19	0.13	-0.43	0.33	0.07	-0.05	-0.19	-0.04	-0.21	-0.17	-0.04	-0.06	0.07	0.14	-0.24	-0.14	-0.14	0.20	0.09	0.09	0.10	-0.15	0.04	-0.07	-0.24	-0.04	0.09	-0.14	-0.03	0.01	-0.19
Proline	0.42	0.20	-0.10	0.09	-0.36	0.22	-0.05	-0.19	1.00	-0.35	0.28	-0.03	-0.20	-0.10		0.28	-0.25	0.09	-0.20	0.15	-0.25	-0.21	-0.23	-0.08	0.02	0.01	0.12	0.01	-0.23	0.20	0.15	-0.08	0.07	0.27	0.25	0.15	0.34	-0.19	0.35
Glycine	-0.25	-0.06	-0.25	-0.33	0.43	-0.28	-0.01	0.13	-0.35	1.00	-0.02	0.14	-0.14	-0.53	-0.47	-0.54	-0.22	-0.21	-0.49	-0.43	-0.14	-0.05	0.01	0.33	0.03	-0.20	0.10	-0.16	0.53	-0.01	0.11	-0.10	-0.21	-0.36	-0.16	-0.33	0.10	0.20	-0.10
Alanine	0.33	0.01	-0.06	-0.06	-0.15	0.37	0.03	-0.43	0.28	-0.02	1.00	-0.16	-0.46	-0.46	0.15	-0.22	-0.05	0.07	-0.26	0.03	-0.32	-0.53	-0.01	-0.15	0.15	-0.10	-0.09	-0.07	-0.07	0.60	0.04	-0.32		0.21	-0.04	0.17	0.18	0.11	0.14
Citrulline	-0.42	0.10	-0.25	-0.18	-0.14	-0.31	-0.27	0.33	-0.03	0.14	-0.16	1.00	0.13	-0.10	-0.22	-0.08	-0.23	0.09	0.05	-0.09	0.15	0.06	-0.07	-0.19	-0.08	-0.03	0.19	0.01	-0.06	-0.20	0.22	0.01	-0.07	-0.15	0.05	-0.08	0.05	-0.28	-0.01
alpha_Aminobutyric_acid	-0.21	0.34	0.10	0.08	0.14	-0.03	-0.15	0.07	-0.20	-0.14	-0.46	0.13	1.00	0.32	0.15	0.23	0.01	-0.08	0.45	0.19	0.30	0.21	-0.03	0.19	0.15	-0.07	0.26	0.04	-0.23	-0.57	0.01	0.81	0.04	0.16	0.05	0.12	-0.50	-0.23	0.04
Valine	0.04	-0.16	-0.11	0.16	-0.26	0.05	-0.12	-0.05	-0.10	-0.53	-0.46	-0.10	0.32	1.00	0.32	0.69	0.36	0.09	0.48	0.17	0.36	0.41	0.11	-0.16	-0.05	0.01	-0.01	0.17	-0.07	-0.25	0.04	0.28	0.28	0.18	0.18	0.20	-0.23	-0.05	0.03
Methionine	0.44	0.07	-0.22	0.34	-0.10	0.40	-0.26	-0.19		-0.47	0.15	-0.22	0.15	0.32	1.00	0.38	0.05	0.15	0.30	0.29	0.05	-0.04	-0.17		0.07	-0.04	0.12	-0.16	-0.20	0.23	0.11	0.16	-0.16	0.66	-0.13	0.15	-0.16	0.15	0.07
Tyrosine	0.18	-0.18	-0.18	0.09	-0.32	0.13	-0.21	-0.04	0.28	-0.54	-0.22	-0.08	0.23	0.69	0.38	1.00	0.04	0.06	0.24	0.31	0.17	0.20	-0.02	0.02	0.08	-0.16	0.16	0.15	-0.08	-0.13	0.16	0.24	0.01	0.27	0.54	0.03	0.10	0.09	0.10
Phenylalanine		-0.04	0.15	-0.02	0.14	0.05	0.40	-0.21	-0.25	-0.22	-0.05	-0.23	0.01	0.36	0.05	0.04	1.00	0.22	0.13	-0.15	0.19	0.03	0.27	-0.12	-0.10	0.10	-0.25	0.29	-0.13	-0.14	-0.02	0.02	0.22	-0.13	0.10	0.58	-0.10	-0.10	-0.02
Orithine	0.09	0.03	-0.11	0.12	0.06	0.14	0.22	-0.17	0.09	-0.21	0.07	0.09	-0.08	0.09	0.15	0.06	0.22	1.00	-0.03	-0.13	-0.07	-0.21	-0.12	-0.01	0.06	0.21	-0.06	0.08	-0.25	0.19	0.18	-0.13	-0.17	0.05	-0.07	0.19	0.27	-0.14	-0.12
Lysine		-0.07	0.01	0.37	-0.24	0.01	-0.24	-0.04	-0.20	-0.49	-0.26	0.05	0.45	0.48	0.30	0.24	0.13	-0.03	1.00	0.31	0.49	0.19	0.16	-0.21	-0.07	0.18	-0.23	0.17	-0.04	-0.29	-0.20	0.31	0.26	0.21	-0.04	0.30	-0.50	-0.06	-0.06
Histidine		-0.08	0.23	0.43	-0.42	0.08	-0.22	-0.06	0.15	-0.43	0.03	-0.09	0.19	0.17	0.29	0.31	-0.15	-0.13	0.31	1.00	0.37	-0.03	-0.04	-0.08	0.07	0.12	-0.07	0.16	-0.17	-0.07	-0.12	0.17	0.09	0.18	0.17	0.04	0.13	0.07	0.36
3Methylhistidine	-0.39	-0.22	0.08	0.34	-0.14	-0.37	-0.22	0.07	-0.25	-0.14	-0.32	0.15	0.30	0.36	0.05	0.17	0.19	-0.07	0.49	0.37	1.00	0.10	0.38	-0.18	-0.21	0.06	-0.23	0.27	0.14	-0.37	0.11	0.21	0.13	-0.09	0.10	0.13	-0.28	-0.12	0.07
1Methylhistidine	-0.04		-0.05	-0.14	0.10	-0.04	-0.23	0.14	-0.21	-0.05	-0.53	0.06	0.21	0.41	-0.04	0.20	0.03	-0.21	0.19	-0.03	0.10	1.00	0.11	0.21	0.05	-0.08	0.04	-0.17	0.16	-0.32	0.11	0.30	0.15	-0.13	-0.06	-0.08	-0.15	-0.03	-0.02
HM_Taurine	-0.31	-0.53	0.25	0.06	0.20	-0.09	0.22	-0.24	-0.23	0.01	-0.01	-0.07	-0.03	0.11	-0.17	-0.02	0.27	-0.12	0.16	-0.04	0.38	0.11	1.00	-0.09	-0.05	-0.23	-0.56	0.47	0.37	-0.21	-0.33	-0.11	0.41	-0.36	0.22	0.10	-0.20	0.16	-0.17
HM_Serine	0.15	0.28	-0.16	-0.10	0.59	0.05	-0.01	-0.14	-0.08	0.33	-0.15	-0.19	0.19	-0.16		0.02	-0.12	-0.01	-0.21	-0.08	-0.18	0.21	-0.09	1.00	0.40	-0.55	0.42	-0.16	0.24	-0.08	0.02	0.49	-0.27	-0.03	-0.06	-0.26	0.15	0.19	-0.06
HM_Asparagine	0.14	0.20	-0.09	-0.07	0.11	0.57	-0.21	-0.14	0.02	0.03	0.15	-0.08	0.15	-0.05	0.07	0.08	-0.10	0.06	-0.07	0.07	-0.21	0.05	-0.05	0.40	1.00	-0.27	0.22	-0.09		-0.04	-0.17	0.18	-0.29	0.07	-0.14	-0.15	0.09	0.17	-0.22
HM_Glutamic_acid	-0.02	0.07	0.08	0.11	-0.04	-0.05	0.07	0.20	0.01	-0.20	-0.10	-0.03	-0.07	0.01	-0.04	-0.16	0.10	0.21	0.18	0.12	0.06	-0.08	-0.23	-0.55	-0.27	1.00	-0.53	-0.02	-0.33	-0.11	-0.24	-0.43	0.05	0.07	-0.13	0.13	-0.15	-0.37	-0.11
HM_Glutamine	0.17	0.49	-0.17	-0.24	-0.11	0.01	-0.15	0.09	0.12	0.10	-0.09	0.19	0.26	-0.01	0.12	0.16	-0.25	-0.06	-0.23	-0.07	-0.23	0.04	-0.56	0.42	0.22	-0.53	1.00	-0.25	-0.19	-0.09	0.43	0.51	-0.43	-0.02	-0.14	-0.28	0.13	-0.13	0.10
HM_Proline	-0.47	-0.30	0.43	0.08	-0.15	-0.14	0.12	0.09	0.01	-0.16	-0.07	0.01	0.04	0.17	-0.16	0.15	0.29	0.08	0.17	0.16	0.27	-0.17	0.47	-0.16	-0.09	-0.02	-0.25	1.00	0.22	-0.37	-0.19	0.02	0.11	-0.27	0.30	0.21	0.01	-0.10	-0.12
HM_Glycine	-0.22	-0.42	-0.19	0.07	0.08	-0.20	-0.21	0.10	-0.23	0.53	-0.07	-0.06	-0.23	-0.07	-0.20	-0.08	-0.13	-0.25	-0.04	-0.17	0.14	0.16	0.37	0.24		-0.33	-0.19	0.22	1.00		-0.02	-0.06	-0.06	-0.15	0.07	-0.19	0.07	0.43	-0.17
HM_Alanine	0.38	-0.22	-0.19	0.06	-0.22	0.25	-0.02	-0.15	0.20	-0.01	0.60	-0.20	-0.57	-0.25	0.23	-0.13	-0.14	0.19	-0.29	-0.07	-0.37	-0.32	-0.21	-0.08	-0.04	-0.11	-0.09	-0.37		1.00	0.22	-0.36	-0.08	0.32	-0.06	0.05	0.42	0.32	0.25
HM_Citrulline	-0.01	0.06	-0.19	-0.11	-0.24	-0.09	-0.17	0.04	0.15	0.11	0.04	0.22	0.01	0.04	0.11	0.16	-0.02	0.18	-0.20	-0.12	0.11	0.11	-0.33	0.02	-0.17	-0.24	0.43	-0.19	-0.02	0.22	1.00	0.24	-0.15	0.08	0.12	0.11	0.20		0.40
HM_alpha_Aminobutyric_acid	-0.08	0.39	0.07	0.04	0.07	-0.03	-0.09	-0.07	-0.08	-0.10	-0.32	0.01	0.81	0.28	0.16	0.24	0.02	-0.13	0.31	0.17	0.21	0.30	-0.11	0.49	0.18	-0.43	0.51	0.02	-0.06	-0.36	0.24	1.00	0.04	0.12	0.10	0.07	-0.29	-0.11	0.12
HM_Valine	-0.14	-0.29	0.12	0.11	-0.04	-0.01	0.15	-0.24	0.07	-0.21		-0.07	0.04	0.28	-0.16	0.01	0.22	-0.17	0.26	0.09	0.13	0.15	0.41	-0.27	-0.29	0.05	-0.43	0.11	-0.06	-0.08	-0.15	0.04	1.00	0.14	0.43	0.47	-0.20	0.10	0.31
HM_Methionine	0.42	0.22	-0.24	0.31	-0.19	0.44	-0.38	-0.04	0.27	-0.36	0.21	-0.15	0.16	0.18	0.66	0.27	-0.13	0.05	0.21	0.18	-0.09	-0.13	-0.36	-0.03	0.07	0.07	-0.02	-0.27	-0.15	0.32	0.08	0.12	0.14	1.00	0.10	0.44	-0.19	0.12	0.12
HM_Tyrosine	-0.29	-0.35	0.10	-0.07	-0.10	-0.06	0.02	0.09	0.25	-0.16	-0.04	0.05	0.05	0.18	-0.13	0.54	0.10	-0.07	-0.04	0.17	0.10	-0.06	0.22	-0.06	-0.14	-0.13	-0.14	0.30	0.07	-0.06	0.12	0.10	0.43	0.10	1.00	0.27	0.23	0.08	0.25
HM_Pheylalanine	0.12	0.04	0.13	0.17	-0.21	0.22	-0.03	-0.14	0.15	-0.33	0.17	-0.08	0.12	0.20	0.15	0.03	0.58	0.19	0.30	0.04	0.13	-0.08	0.10	-0.26	-0.15	0.13	-0.28	0.21	-0.19	0.05	0.11	0.07	0.47	0.44	0.27	1.00	-0.19	-0.09	0.21
HM_Ornithine	0.08	-0.23	-0.15	-0.02	-0.13	0.07	0.04	-0.03	0.34	0.10	0.18	0.05	-0.50	-0.23	-0.16	0.10	-0.10	0.27	-0.50	0.13	-0.28	-0.15	-0.20	0.15	0.09	-0.15	0.13	0.01	0.07	0.42	0.20	-0.29	-0.20	-0.19	0.23	-0.19	1.00	0.22	0.37
HM_Lysine	0.02	-0.44	-0.05	0.13	0.02	0.14	-0.08	0.01	-0.19	0.20	0.11	-0.28	-0.23	-0.05	0.15	0.09	-0.10	-0.14	-0.06	0.07	-0.12	-0.03	0.16	0.19	0.17	-0.37	-0.13	-0.10	0.43	0.32		-0.11	0.10	0.12	0.08	-0.09	0.22	1.00	0.22
HM_Histidine	0.06	-0.04	-0.10	0.25	-0.24	-0.05	-0.07	-0.19	0.35	-0.10	0.14	-0.01	0.04	0.03	0.07	0.10	-0.02	-0.12	-0.06	0.36	0.07	-0.02	-0.17	-0.06	-0.22	-0.11	0.10	-0.12	-0.17	0.25	0.40	0.12	0.31	0.12	0.25	0.21	0.37	0.22	1.00

online resource 2b

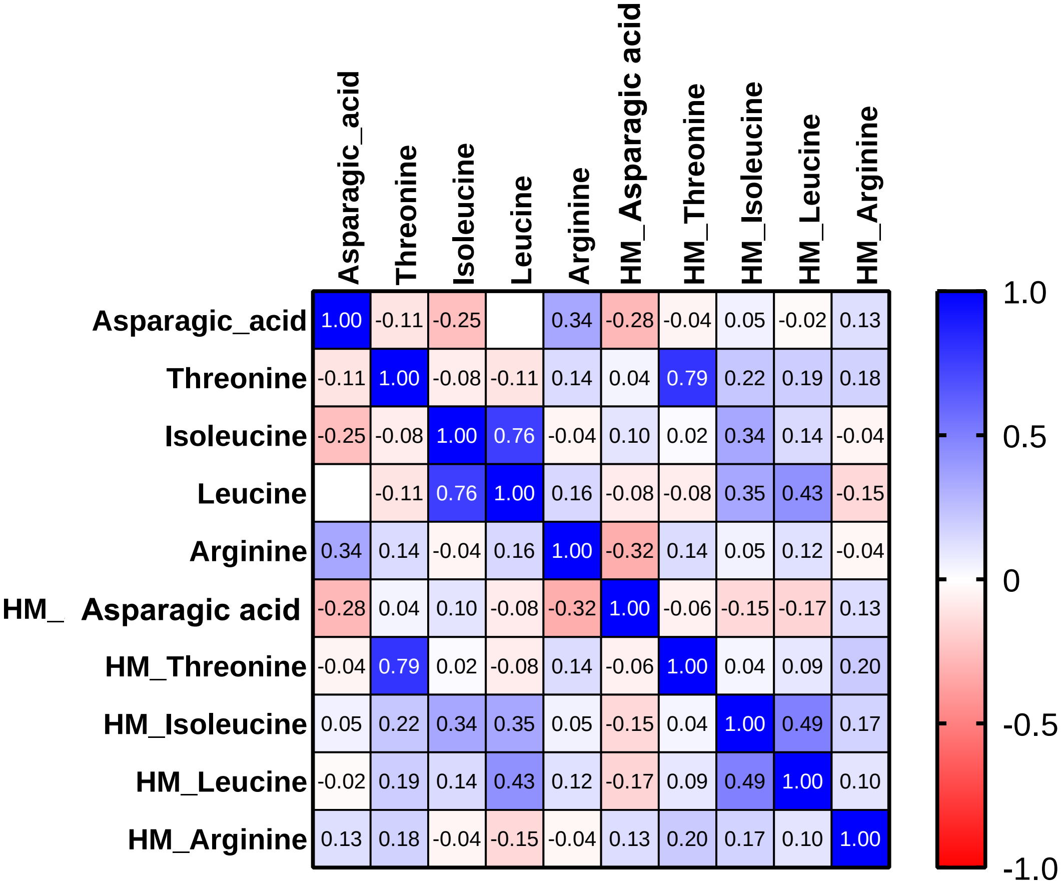

Online resource 3: Microbiota compositon in infant faecal samples;
3a Overview, showing only the most abundant entities >10%;

Phyla	N	Mean Abundance in %	Std. Deviation
D_1__Firmicutes	41	36.4	16.1
D_1__Actinobacteria	41	26	15.9
D_1__Proteobacteria	41	21.7	10.8
D_1__Bacteroidetes	41	15.7	19.7
Classes			
D_0__Bacteria;D_1__Actinobacteria;D_2__Actinobacteria	41	25.7	15.9
D_0__Bacteria;D_1__Proteobacteria;D_2__Gammaproteobacteria	41	21.5	11
D_0__Bacteria;D_1__Bacteroidetes;D_2__Bacteroidia	41	15.7	19.7
D_0__Bacteria;D_1__Firmicutes;D_2__Clostridia	41	15.3	15
D_0__Bacteria;D_1__Firmicutes;D_2__Bacilli	41	13.5	10.1
Orders			
D_2__Actinobacteria;D_3__Bifidobacteriales	41	23.6	15.5
D_2__Gammaproteobacteria;D_3__Enterobacteriales	41	19	10.6
D_2__Bacteroidia;D_3__Bacteroidales	41	15.7	19.7
D_1__Firmicutes;D_2__Clostridia;D_3__Clostridiales	41	15.3	15
D_1__Firmicutes;D_2__Bacilli;D_3__Lactobacillales	41	11.9	8.5
Families			
D_4__Bifidobacteriaceae	41	23.6	15.5
D_4__Enterobacteriaceae	41	19	10.6
D_4__Bacteroidaceae	41	11.9	17
D_4__Clostridiaceae 1	41	10.8	13.2
Genera			
D_4__Bifidobacteriaceae;D_5__Bifidobacterium	41	23.6	15.6
D_4__Enterobacteriaceae;D_5__Escherichia-Shigella	41	13.3	13.6
D_4__Bacteroidaceae;D_5__Bacteroides	41	11.9	17
D_4__Clostridiaceae 1;D_5__Clostridium sensu stricto 1	41	10.8	13.2

online resource 3b: Microbiota composition of infant faecal samples (all entities)

**phyla**	N	Mean Abundance	Std. Deviation
D_0__Bacteria;D_1__Firmicutes	41	0.363576322	0.160720978
D_0__Bacteria;D_1__Actinobacteria	41	0.25989542	0.159133967
D_0__Bacteria;D_1__Proteobacteria	41	0.217267767	0.107520741
D_0__Bacteria;D_1__Bacteroidetes	41	0.156560357	0.196553055
D_0__Bacteria;D_1__Verrucomicrobia	41	0.001280909	0.007997839
D_0__Bacteria;D_1__Epsilonbacteraeota	41	0.000755532	0.004837764
D_0__Bacteria;D_1__Fusobacteria	41	0.000332878	0.000979205
D_0__Archaea;D_1__Euryarchaeota	41	0.000184101	0.000492383
D_0__Bacteria;Other	41	9.31195E-05	0.000187799
D_0__Bacteria;D_1__Cyanobacteria	41	3.35659E-05	0.000191621
D_0__Bacteria;D_1__Lentisphaerae	41	8.40793E-06	3.7932E-05
D_0__Bacteria;D_1__Tenericutes	41	8.20699E-06	3.68202E-05
D_0__Bacteria;D_1__Elusimicrobia	41	3.41313E-06	2.18547E-05

**classes**	N	Mean Abundance	Std. Deviation
D_0__Bacteria;D_1__Actinobacteria;D_2__Actinobacteria	41	0.256724432	0.158803799
D_0__Bacteria;D_1__Proteobacteria;D_2__Gammaproteobacteria	41	0.214570376	0.109974456
D_0__Bacteria;D_1__Bacteroidetes;D_2__Bacteroidia	41	0.156560357	0.196553055
D_0__Bacteria;D_1__Firmicutes;D_2__Clostridia	41	0.153147901	0.149814653
D_0__Bacteria;D_1__Firmicutes;D_2__Bacilli	41	0.134761973	0.101007126
D_0__Bacteria;D_1__Firmicutes;D_2__Negativicutes	41	0.067010454	0.073275135
D_0__Bacteria;D_1__Firmicutes;D_2__Erysipelotrichia	41	0.008634962	0.025278601
D_0__Bacteria;D_1__Actinobacteria;D_2__Coriobacteriia	41	0.003166468	0.007192438
D_0__Bacteria;D_1__Proteobacteria;D_2__Alphaproteobacteria	41	0.001349085	0.007356778
D_0__Bacteria;D_1__Proteobacteria;D_2__Deltaproteobacteria	41	0.00134404	0.008066887
D_0__Bacteria;D_1__Verrucomicrobia;D_2__Verrucomicrobiae	41	0.001280909	0.007997839
D_0__Bacteria;D_1__Epsilonbacteraeota;D_2__Campylobacteria	41	0.000755532	0.004837764
D_0__Bacteria;D_1__Fusobacteria;D_2__Fusobacteriia	41	0.000332878	0.000979205
D_0__Archaea;D_1__Euryarchaeota;D_2__Methanobacteria	41	0.000182395	0.000489421
D_0__Bacteria;Other;Other	41	9.31195E-05	0.000187799
D_0__Bacteria;D_1__Cyanobacteria;D_2__Melainabacteria	41	2.97805E-05	0.000190688
D_0__Bacteria;D_1__Firmicutes;Other	41	2.10318E-05	0.000127497
D_0__Bacteria;D_1__Lentisphaerae;D_2__Lentisphaeria	41	8.40793E-06	3.7932E-05
D_0__Bacteria;D_1__Tenericutes;D_2__Mollicutes	41	8.20699E-06	3.68202E-05
D_0__Bacteria;D_1__Actinobacteria;Other	41	4.51995E-06	2.30023E-05
D_0__Bacteria;D_1__Proteobacteria;Other	41	4.26642E-06	2.73184E-05
D_0__Bacteria;D_1__Cyanobacteria;D_2__Oxyphotobacteria	41	3.78542E-06	2.42385E-05
D_0__Bacteria;D_1__Elusimicrobia;D_2__Elusimicrobia	41	3.41313E-06	2.18547E-05
D_0__Archaea;D_1__Euryarchaeota;D_2__Thermoplasmata	41	1.70657E-06	1.09274E-05

**Orders**	N	Mean Abundance	Std. Deviation
D_0__Bacteria;D_1__Actinobacteria;D_2__Actinobacteria;D_3__Bifidobacteriales	41	0.236370291	0.155398044
D_2__Gammaproteobacteria;D_3__Enterobacteriales	41	0.190022889	0.106338399
D_0__Bacteria;D_1__Bacteroidetes;D_2__Bacteroidia;D_3__Bacteroidales	41	0.15655764	0.196549814
D_0__Bacteria;D_1__Firmicutes;D_2__Clostridia;D_3__Clostridiales	41	0.153147901	0.149814653
D_0__Bacteria;D_1__Firmicutes;D_2__Bacilli;D_3__Lactobacillales	41	0.119319105	0.084574636
D_0__Bacteria;D_1__Firmicutes;D_2__Negativicutes;D_3__Selenomonadales	41	0.067010454	0.073275135
D_0__Bacteria;D_1__Proteobacteria;D_2__Gammaproteobacteria;D_3__Pasteurellales	41	0.023177499	0.054680794
D_0__Bacteria;D_1__Firmicutes;D_2__Bacilli;D_3__Bacillales	41	0.015442867	0.033454462
D_0__Bacteria;D_1__Actinobacteria;D_2__Actinobacteria;D_3__Micrococcales	41	0.014010016	0.024162614
D_0__Bacteria;D_1__Firmicutes;D_2__Erysipelotrichia;D_3__Erysipelotrichales	41	0.008634962	0.025278601
D_0__Bacteria;D_1__Actinobacteria;D_2__Actinobacteria;D_3__Actinomycetales	41	0.00416529	0.012976725
D_0__Bacteria;D_1__Actinobacteria;D_2__Coriobacteriia;D_3__Coriobacteriales	41	0.003166468	0.007192438
D_0__Bacteria;D_1__Actinobacteria;D_2__Actinobacteria;D_3__Propionibacteriales	41	0.001829291	0.005045055
D_0__Bacteria;D_1__Proteobacteria;D_2__Deltaproteobacteria;D_3__Desulfovibrionales	41	0.00134404	0.008066887
D_0__Bacteria;D_1__Proteobacteria;D_2__Alphaproteobacteria;D_3__Rhodospirillales	41	0.001306135	0.007359453
D_0__Bacteria;D_1__Verrucomicrobia;D_2__Verrucomicrobiae;D_3__Verrucomicrobiales	41	0.001280909	0.007997839
D_0__Bacteria;D_1__Proteobacteria;D_2__Gammaproteobacteria;D_3__Betaproteobacteriales	41	0.001279592	0.00429056
D_0__Bacteria;D_1__Epsilonbacteraeota;D_2__Campylobacteria;D_3__Campylobacterales	41	0.000755532	0.004837764
D_0__Bacteria;D_1__Actinobacteria;D_2__Actinobacteria;D_3__Corynebacteriales	41	0.000341106	0.000770698
D_0__Bacteria;D_1__Fusobacteria;D_2__Fusobacteriia;D_3__Fusobacteriales	41	0.000332878	0.000979205
D_0__Archaea;D_1__Euryarchaeota;D_2__Methanobacteria;D_3__Methanobacteriales	41	0.000182395	0.000489421
D_0__Bacteria;Other;Other;Other	41	9.31195E-05	0.000187799
D_0__Bacteria;D_1__Proteobacteria;D_2__Gammaproteobacteria;Other	41	5.01172E-05	0.000188698
D_0__Bacteria;D_1__Proteobacteria;D_2__Alphaproteobacteria;D_3__Rhodobacterales	41	3.55977E-05	0.000227937
D_0__Bacteria;D_1__Proteobacteria;D_2__Gammaproteobacteria;D_3__Pseudomonadales	41	3.44742E-05	0.00015684
D_0__Bacteria;D_1__Cyanobacteria;D_2__Melainabacteria;D_3__Gastranaerophilales	41	2.97805E-05	0.000190688
D_0__Bacteria;D_1__Firmicutes;Other;Other	41	2.10318E-05	0.000127497
D_0__Bacteria;D_1__Actinobacteria;D_2__Actinobacteria;Other	41	8.43898E-06	5.40359E-05
D_0__Bacteria;D_1__Lentisphaerae;D_2__Lentisphaeria;D_3__Victivallales	41	8.40793E-06	3.7932E-05
D_0__Bacteria;D_1__Tenericutes;D_2__Mollicutes;D_3__Izimaplasmatales	41	8.20699E-06	3.68202E-05
D_0__Bacteria;D_1__Proteobacteria;D_2__Alphaproteobacteria;D_3__Rhizobiales	41	7.3517E-06	4.70739E-05
D_0__Bacteria;D_1__Proteobacteria;D_2__Gammaproteobacteria;D_3__Xanthomonadales	41	5.80398E-06	3.71636E-05
D_0__Bacteria;D_1__Actinobacteria;Other;Other	41	4.51995E-06	2.30023E-05
D_0__Bacteria;D_1__Proteobacteria;Other;Other	41	4.26642E-06	2.73184E-05
D_0__Bacteria;D_1__Cyanobacteria;D_2__Oxyphotobacteria;D_3__Chloroplast	41	3.78542E-06	2.42385E-05
D_0__Bacteria;D_1__Elusimicrobia;D_2__Elusimicrobia;D_3__Elusimicrobiales	41	3.41313E-06	2.18547E-05
D_0__Bacteria;D_1__Bacteroidetes;D_2__Bacteroidia;D_3__Flavobacteriales	41	2.71764E-06	1.22727E-05
D_0__Archaea;D_1__Euryarchaeota;D_2__Thermoplasmata;D_3__Methanomassiliicoccales	41	1.70657E-06	1.09274E-05
D_0__Bacteria;D_1__Firmicutes;D_2__Bacilli;Other	41	0	0

**families**	N	Mean Abundance	Std. Deviation
D_0__Bacteria;D_1__Actinobacteria;D_2__Actinobacteria;D_3__Bifidobacteriales;D_4__Bifidobacteriaceae	41	0.236370291	0.155398044
D_0__Bacteria;D_1__Proteobacteria;D_2__Gammaproteobacteria;D_3__Enterobacteriales;D_4__Enterobacteriaceae	41	0.190022889	0.106338399
D_0__Bacteria;D_1__Bacteroidetes;D_2__Bacteroidia;D_3__Bacteroidales;D_4__Bacteroidaceae	41	0.119439767	0.169844824
D_0__Bacteria;D_1__Firmicutes;D_2__Clostridia;D_3__Clostridiales;D_4__Clostridiaceae 1	41	0.107718739	0.131667966
D_0__Bacteria;D_1__Firmicutes;D_2__Bacilli;D_3__Lactobacillales;D_4__Streptococcaceae	41	0.081448571	0.078433627
D_0__Bacteria;D_1__Firmicutes;D_2__Negativicutes;D_3__Selenomonadales;D_4__Veillonellaceae	41	0.066977696	0.073289392
D_0__Bacteria;D_1__Firmicutes;D_2__Clostridia;D_3__Clostridiales;D_4__Lachnospiraceae	41	0.026274661	0.047925786
D_0__Bacteria;D_1__Proteobacteria;D_2__Gammaproteobacteria;D_3__Pasteurellales;D_4__Pasteurellaceae	41	0.023177499	0.054680794
D_0__Bacteria;D_1__Firmicutes;D_2__Bacilli;D_3__Lactobacillales;D_4__Enterococcaceae	41	0.021397875	0.044032311
D_0__Bacteria;D_1__Bacteroidetes;D_2__Bacteroidia;D_3__Bacteroidales;D_4__Prevotellaceae	41	0.01778571	0.05774052
D_0__Bacteria;D_1__Firmicutes;D_2__Bacilli;D_3__Lactobacillales;D_4__Lactobacillaceae	41	0.016120826	0.037740877
D_0__Bacteria;D_1__Bacteroidetes;D_2__Bacteroidia;D_3__Bacteroidales;D_4__Tannerellaceae	41	0.015925824	0.041957899
D_0__Bacteria;D_1__Firmicutes;D_2__Bacilli;D_3__Bacillales;D_4__Staphylococcaceae	41	0.014400671	0.032604453
D_0__Bacteria;D_1__Actinobacteria;D_2__Actinobacteria;D_3__Micrococcales;D_4__Micrococcaceae	41	0.013962548	0.024156365
D_0__Bacteria;D_1__Firmicutes;D_2__Clostridia;D_3__Clostridiales;D_4__Ruminococcaceae	41	0.012994187	0.028067757
D_0__Bacteria;D_1__Firmicutes;D_2__Erysipelotrichia;D_3__Erysipelotrichales;D_4__Erysipelotrichaceae	41	0.008634962	0.025278601
D_0__Bacteria;D_1__Firmicutes;D_2__Clostridia;D_3__Clostridiales;D_4__Peptostreptococcaceae	41	0.006049476	0.016497695
D_0__Bacteria;D_1__Actinobacteria;D_2__Actinobacteria;D_3__Actinomycetales;D_4__Actinomycetaceae	41	0.00416529	0.012976725
D_0__Bacteria;D_1__Actinobacteria;D_2__Coriobacteriia;D_3__Coriobacteriales;D_4__Eggerthellaceae	41	0.002934243	0.007211226
D_0__Bacteria;D_1__Bacteroidetes;D_2__Bacteroidia;D_3__Bacteroidales;D_4__Rikenellaceae	41	0.002559365	0.008452785
D_0__Bacteria;D_1__Actinobacteria;D_2__Actinobacteria;D_3__Propionibacteriales;D_4__Propionibacteriaceae	41	0.001829291	0.005045055

D_0__Bacteria;D_1__Proteobacteria;D_2__Deltaproteobacteria;D_3__Desulfovibrionales;D_4__Desulfovibrionaceae	41	0.00134404	0.008066887
D_0__Bacteria;D_1__Proteobacteria;D_2__Alphaproteobacteria;D_3__Rhodospirillales;D_4__uncultured	41	0.001306135	0.007359453
D_0__Bacteria;D_1__Verrucomicrobia;D_2__Verrucomicrobiae;D_3__Verrucomicrobiales;D_4__Akkermansiaceae	41	0.001280909	0.007997839
D_0__Bacteria;D_1__Proteobacteria;D_2__Gammaproteobacteria;D_3__Betaproteobacteriales;D_4__Burkholderiaceae	41	0.001255445	0.004296112
D_0__Bacteria;D_1__Firmicutes;D_2__Bacilli;D_3__Bacillales;D_4__Family XI	41	0.00101693	0.002640536
D_0__Bacteria;D_1__Epsilonbacteraeota;D_2__Campylobacteria;D_3__Campylobacterales;D_4__Campylobacteraceae	41	0.000755532	0.004837764
D_0__Bacteria;D_1__Bacteroidetes;D_2__Bacteroidia;D_3__Bacteroidales;D_4__Marinifilaceae	41	0.00074863	0.002498784
D_0__Bacteria;D_1__Actinobacteria;D_2__Actinobacteria;D_3__Corynebacteriales;D_4__Corynebacteriaceae	41	0.000341106	0.000770698
D_0__Bacteria;D_1__Fusobacteria;D_2__Fusobacteriia;D_3__Fusobacteriales;D_4__Fusobacteriaceae	41	0.000332878	0.000979205
D_0__Bacteria;D_1__Firmicutes;D_2__Bacilli;D_3__Lactobacillales;D_4__Carnobacteriaceae	41	0.000287202	0.001706829
D_0__Bacteria;D_1__Actinobacteria;D_2__Coriobacteriia;D_3__Coriobacteriales;D_4__Atopobiaceae	41	0.000230539	0.000788914
D_0__Archaea;D_1__Euryarchaeota;D_2__Methanobacteria;D_3__Methanobacteriales;D_4__Methanobacteriaceae	41	0.000182395	0.000489421
D_0__Bacteria;Other;Other;Other;Other	41	9.31195E-05	0.000187799
D_0__Bacteria;D_1__Bacteroidetes;D_2__Bacteroidia;D_3__Bacteroidales;D_4__Barnesiellaceae	41	6.42518E-05	0.000228095
D_0__Bacteria;D_1__Firmicutes;D_2__Bacilli;D_3__Lactobacillales;Other	41	5.93131E-05	0.000270351
D_0__Bacteria;D_1__Proteobacteria;D_2__Gammaproteobacteria;Other;Other	41	5.01172E-05	0.000188698
D_0__Bacteria;D_1__Firmicutes;D_2__Clostridia;D_3__Clostridiales;D_4__Family XI	41	4.80269E-05	0.000207135
D_0__Bacteria;D_1__Firmicutes;D_2__Clostridia;D_3__Clostridiales;D_4__Christensenellaceae	41	4.34196E-05	0.000224727
D_0__Bacteria;D_1__Actinobacteria;D_2__Actinobacteria;D_3__Micrococcales;D_4__Dermabacteraceae	41	4.18037E-05	0.000267675
D_0__Bacteria;D_1__Proteobacteria;D_2__Alphaproteobacteria;D_3__Rhodobacterales;D_4__Rhodobacteraceae	41	3.55977E-05	0.000227937
D_0__Bacteria;D_1__Bacteroidetes;D_2__Bacteroidia;D_3__Bacteroidales;D_4__Muribaculaceae	41	3.40926E-05	0.000198505
D_0__Bacteria;D_1__Firmicutes;D_2__Negativicutes;D_3__Selenomonadales;D_4__Acidaminococcaceae	41	3.27574E-05	0.00020975
D_0__Bacteria;D_1__Proteobacteria;D_2__Gammaproteobacteria;D_3__Pseudomonadales;D_4__Moraxellaceae	41	3.0218E-05	0.000131144
D_0__Bacteria;D_1__Cyanobacteria;D_2__Melainabacteria;D_3__Gastranaerophilales;Other	41	2.97805E-05	0.000190688
D_0__Bacteria;D_1__Firmicutes;D_2__Bacilli;D_3__Bacillales;D_4__Paenibacillaceae	41	2.52666E-05	0.000161785
D_0__Bacteria;D_1__Proteobacteria;D_2__Gammaproteobacteria;D_3__Betaproteobacteriales;D_4__Neisseriaceae	41	2.41472E-05	0.000120309
D_0__Bacteria;D_1__Firmicutes;Other;Other;Other	41	2.10318E-05	0.000127497
D_0__Bacteria;D_1__Firmicutes;D_2__Clostridia;D_3__Clostridiales;D_4__Clostridiales vadinBB60 group	41	9.8188E-06	4.9673E-05
D_0__Bacteria;D_1__Actinobacteria;D_2__Actinobacteria;Other;Other	41	8.43898E-06	5.40359E-05
D_0__Bacteria;D_1__Tenericutes;D_2__Mollicutes;D_3__Izimaplasmatales;D_4__uncultured organism	41	8.20699E-06	3.68202E-05
D_0__Bacteria;D_1__Proteobacteria;D_2__Alphaproteobacteria;D_3__Rhizobiales;D_4__Rhizobiaceae	41	7.3517E-06	4.70739E-05
D_0__Bacteria;D_1__Proteobacteria;D_2__Gammaproteobacteria;D_3__Xanthomonadales;D_4__Xanthomonadaceae	41	5.80398E-06	3.71636E-05
D_0__Bacteria;D_1__Actinobacteria;D_2__Actinobacteria;D_3__Micrococcales;D_4__Dermatophilaceae	41	5.66359E-06	3.62647E-05
D_0__Bacteria;D_1__Firmicutes;D_2__Clostridia;D_3__Clostridiales;D_4__Defluviitaleaceae	41	5.31795E-06	3.40515E-05
D_0__Bacteria;D_1__Firmicutes;D_2__Bacilli;D_3__Lactobacillales;D_4__Leuconostocaceae	41	5.31795E-06	3.40515E-05
D_0__Bacteria;D_1__Lentisphaerae;D_2__Lentisphaeria;D_3__Victivallales;D_4__vadinBE97	41	4.75861E-06	3.047E-05
D_0__Bacteria;D_1__Actinobacteria;Other;Other;Other	41	4.51995E-06	2.30023E-05
D_0__Bacteria;D_1__Proteobacteria;Other;Other;Other	41	4.26642E-06	2.73184E-05
D_0__Bacteria;D_1__Proteobacteria;D_2__Gammaproteobacteria;D_3__Pseudomonadales;D_4__Pseudomonadaceae	41	4.25625E-06	2.72533E-05
D_0__Bacteria;D_1__Firmicutes;D_2__Clostridia;D_3__Clostridiales;Other	41	4.25436E-06	2.72412E-05
D_0__Bacteria;D_1__Cyanobacteria;D_2__Oxyphotobacteria;D_3__Chloroplast;Other	41	3.78542E-06	2.42385E-05
D_0__Bacteria;D_1__Lentisphaerae;D_2__Lentisphaeria;D_3__Victivallales;D_4__Victivallaceae	41	3.64932E-06	2.33671E-05
D_0__Bacteria;D_1__Elusimicrobia;D_2__Elusimicrobia;D_3__Elusimicrobiales;D_4__Elusimicrobiaceae	41	3.41313E-06	2.18547E-05
D_0__Archaea;D_1__Euryarchaeota;D_2__Thermoplasmata;D_3__Methanomassiliicoccales;D_4__Methanomethylophilaceae	41	1.70657E-06	1.09274E-05
D_0__Bacteria;D_1__Actinobacteria;D_2__Coriobacteriia;D_3__Coriobacteriales;D_4__Coriobacteriales Incertae Sedis	41	1.68561E-06	1.07932E-05
D_0__Bacteria;D_1__Bacteroidetes;D_2__Bacteroidia;D_3__Flavobacteriales;D_4__Weeksellaceae	41	1.54773E-06	9.91029E-06
D_0__Bacteria;D_1__Bacteroidetes;D_2__Bacteroidia;D_3__Flavobacteriales;D_4__Flavobacteriaceae	41	1.16991E-06	7.49107E-06

**genera**	N	Mean Abundance	Std. Deviation
D_0__Bacteria;D_1__Actinobacteria;D_2__Actinobacteria;D_3__Bifidobacteriales;D_4__Bifidobacteriaceae;D_5__Bifidobacterium	41	0.236269584	0.155553821
D_0__Bacteria;D_1__Proteobacteria;D_2__Gammaproteobacteria;D_3__Enterobacteriales;D_4__Enterobacteriaceae;D_5__Escherichia-Shigella	41	0.133433548	0.136018831
D_0__Bacteria;D_1__Bacteroidetes;D_2__Bacteroidia;D_3__Bacteroidales;D_4__Bacteroidaceae;D_5__Bacteroides	41	0.119439767	0.169844824
D_0__Bacteria;D_1__Firmicutes;D_2__Clostridia;D_3__Clostridiales;D_4__Clostridiaceae 1;D_5__Clostridium sensu stricto 1	41	0.107619666	0.131630991
D_0__Bacteria;D_1__Firmicutes;D_2__Bacilli;D_3__Lactobacillales;D_4__Streptococcaceae;D_5__Streptococcus	41	0.081439212	0.078434542
D_0__Bacteria;D_1__Firmicutes;D_2__Negativicutes;D_3__Selenomonadales;D_4__Veillonellaceae;D_5__Veillonella	41	0.064358168	0.074222164
D_0__Bacteria;D_1__Proteobacteria;D_2__Gammaproteobacteria;D_3__Enterobacteriales;D_4__Enterobacteriaceae;Other	41	0.052943157	0.079514867
D_0__Bacteria;D_1__Proteobacteria;D_2__Gammaproteobacteria;D_3__Pasteurellales;D_4__Pasteurellaceae;D_5__Haemophilus	41	0.022814562	0.054091822
D_0__Bacteria;D_1__Firmicutes;D_2__Bacilli;D_3__Lactobacillales;D_4__Enterococcaceae;D_5__Enterococcus	41	0.021389753	0.044032476
D_0__Bacteria;D_1__Firmicutes;D_2__Bacilli;D_3__Lactobacillales;D_4__Lactobacillaceae;D_5__Lactobacillus	41	0.016120826	0.037740877
D_0__Bacteria;D_1__Bacteroidetes;D_2__Bacteroidia;D_3__Bacteroidales;D_4__Tannerellaceae;D_5__Parabacteroides	41	0.015925824	0.041957899
D_0__Bacteria;D_1__Firmicutes;D_2__Bacilli;D_3__Bacillales;D_4__Staphylococcaceae;D_5__Staphylococcus	41	0.014400671	0.032604453
D_0__Bacteria;D_1__Actinobacteria;D_2__Actinobacteria;D_3__Micrococcales;D_4__Micrococcaceae;D_5__Rothia	41	0.013869858	0.024059152
D_0__Bacteria;D_1__Bacteroidetes;D_2__Bacteroidia;D_3__Bacteroidales;D_4__Prevotellaceae;D_5__Prevotella 9	41	0.013007805	0.053262858
D_0__Bacteria;D_1__Firmicutes;D_2__Erysipelotrichia;D_3__Erysipelotrichales;D_4__Erysipelotrichaceae;D_5__Erysipelatoclostridium	41	0.006055189	0.023101724
D_0__Bacteria;D_1__Bacteroidetes;D_2__Bacteroidia;D_3__Bacteroidales;D_4__Prevotellaceae;D_5__Prevotella	41	0.004579933	0.024952526
D_0__Bacteria;D_1__Firmicutes;D_2__Clostridia;D_3__Clostridiales;D_4__Ruminococcaceae;D_5__Faecalibacterium	41	0.004533645	0.011637207
D_0__Bacteria;D_1__Firmicutes;D_2__Clostridia;D_3__Clostridiales;D_4__Lachnospiraceae;D_5__Blautia	41	0.004477559	0.011086283
D_0__Bacteria;D_1__Firmicutes;D_2__Clostridia;D_3__Clostridiales;D_4__Peptostreptococcaceae;D_5__Intestinibacter	41	0.004236411	0.015003398
D_0__Bacteria;D_1__Firmicutes;D_2__Clostridia;D_3__Clostridiales;D_4__Lachnospiraceae;D_5__[Ruminococcus] gnavus group	41	0.004159376	0.021960426
D_0__Bacteria;D_1__Actinobacteria;D_2__Actinobacteria;D_3__Actinomycetales;D_4__Actinomycetaceae;D_5__Actinomyces	41	0.003980082	0.01290888
D_0__Bacteria;D_1__Firmicutes;D_2__Clostridia;D_3__Clostridiales;D_4__Ruminococcaceae;D_5__Ruminiclostridium 5	41	0.003482208	0.020615969
D_0__Bacteria;D_1__Firmicutes;D_2__Clostridia;D_3__Clostridiales;D_4__Lachnospiraceae;D_5__Hungatella	41	0.003182188	0.015014603
D_0__Bacteria;D_1__Proteobacteria;D_2__Gammaproteobacteria;D_3__Enterobacteriales;D_4__Enterobacteriaceae;D_5__Serratia	41	0.002863455	0.017608107
D_0__Bacteria;D_1__Firmicutes;D_2__Clostridia;D_3__Clostridiales;D_4__Lachnospiraceae;D_5__Epulopiscium	41	0.002774991	0.015145289
D_0__Bacteria;D_1__Actinobacteria;D_2__Coriobacteriia;D_3__Coriobacteriales;D_4__Eggerthellaceae;D_5__Eggerthella	41	0.002619046	0.007099871
D_0__Bacteria;D_1__Bacteroidetes;D_2__Bacteroidia;D_3__Bacteroidales;D_4__Rikenellaceae;D_5__Alistipes	41	0.002444525	0.008471864
D_0__Bacteria;D_1__Firmicutes;D_2__Erysipelotrichia;D_3__Erysipelotrichales;D_4__Erysipelotrichaceae;D_5__[Clostridium] innocuum group	41	0.001836367	0.011502588
D_0__Bacteria;D_1__Actinobacteria;D_2__Actinobacteria;D_3__Propionibacteriales;D_4__Propionibacteriaceae;D_5__Cutibacterium	41	0.001829291	0.005045055
D_0__Bacteria;D_1__Firmicutes;D_2__Clostridia;D_3__Clostridiales;D_4__Peptostreptococcaceae;D_5__Clostridioides	41	0.001705223	0.006899667
D_0__Bacteria;D_1__Firmicutes;D_2__Clostridia;D_3__Clostridiales;D_4__Lachnospiraceae;D_5__Agathobacter	41	0.001614847	0.008304999
D_0__Bacteria;D_1__Firmicutes;D_2__Clostridia;D_3__Clostridiales;D_4__Lachnospiraceae;D_5__Lachnospira	41	0.001389268	0.005584724
D_0__Bacteria;D_1__Proteobacteria;D_2__Deltaproteobacteria;D_3__Desulfovibrionales;D_4__Desulfovibrionaceae;D_5__Bilophila	41	0.001330117	0.008066137
D_0__Bacteria;D_1__Verrucomicrobia;D_2__Verrucomicrobiae;D_3__Verrucomicrobiales;D_4__Akkermansiaceae;D_5__Akkermansia	41	0.001280909	0.007997839
D_0__Bacteria;D_1__Proteobacteria;D_2__Alphaproteobacteria;D_3__Rhodospirillales;D_4__uncultured;D_5__gut metagenome	41	0.00123441	0.007345301
D_0__Bacteria;D_1__Firmicutes;D_2__Negativicutes;D_3__Selenomonadales;D_4__Veillonellaceae;D_5__Dialister	41	0.001117648	0.00428532
D_0__Bacteria;D_1__Firmicutes;D_2__Clostridia;D_3__Clostridiales;D_4__Lachnospiraceae;D_5__Anaerostipes	41	0.001117533	0.003005168
D_0__Bacteria;D_1__Firmicutes;D_2__Clostridia;D_3__Clostridiales;D_4__Ruminococcaceae;D_5__uncultured	41	0.001090096	0.005104713
D_0__Bacteria;D_1__Firmicutes;D_2__Clostridia;D_3__Clostridiales;D_4__Lachnospiraceae;Other	41	0.001056285	0.003702074
D_0__Bacteria;D_1__Firmicutes;D_2__Bacilli;D_3__Bacillales;D_4__Family XI;D_5__Gemella	41	0.00101693	0.002640536
D_0__Bacteria;D_1__Firmicutes;D_2__Clostridia;D_3__Clostridiales;D_4__Lachnospiraceae;D_5__Lachnoclostridium	41	0.001009373	0.002534844
D_0__Bacteria;D_1__Proteobacteria;D_2__Gammaproteobacteria;D_3__Betaproteobacteriales;D_4__Burkholderiaceae;D_5__Sutterella	41	0.000931493	0.003637313
D_0__Bacteria;D_1__Firmicutes;D_2__Negativicutes;D_3__Selenomonadales;D_4__Veillonellaceae;D_5__Negativicoccus	41	0.000852455	0.00326465
D_0__Bacteria;D_1__Firmicutes;D_2__Clostridia;D_3__Clostridiales;D_4__Lachnospiraceae;D_5__Anaerosporobacter	41	0.000762172	0.004880281
D_0__Bacteria;D_1__Epsilonbacteraeota;D_2__Campylobacteria;D_3__Campylobacterales;D_4__Campylobacteraceae;D_5__Campylobacter	41	0.000755532	0.004837764
D_0__Bacteria;D_1__Firmicutes;D_2__Erysipelotrichia;D_3__Erysipelotrichales;D_4__Erysipelotrichaceae;D_5__Erysipelotrichaceae UCG-003	41	0.000724215	0.002658715
D_0__Bacteria;D_1__Firmicutes;D_2__Clostridia;D_3__Clostridiales;D_4__Lachnospiraceae;D_5__Roseburia	41	0.000687639	0.002057325
D_0__Bacteria;D_1__Firmicutes;D_2__Clostridia;D_3__Clostridiales;D_4__Ruminococcaceae;D_5__Ruminococcus 1	41	0.000666549	0.002173807
D_0__Bacteria;D_1__Bacteroidetes;D_2__Bacteroidia;D_3__Bacteroidales;D_4__Marinifilaceae;D_5__Odoribacter	41	0.000605941	0.00217368
D_0__Bacteria;D_1__Firmicutes;D_2__Clostridia;D_3__Clostridiales;D_4__Ruminococcaceae;D_5__Ruminococcus 2	41	0.000564975	0.002153857
D_0__Bacteria;D_1__Firmicutes;D_2__Clostridia;D_3__Clostridiales;D_4__Ruminococcaceae;D_5__UBA1819	41	0.000530974	0.003315548
D_0__Bacteria;D_1__Firmicutes;D_2__Clostridia;D_3__Clostridiales;D_4__Ruminococcaceae;D_5__Subdoligranulum	41	0.000486228	0.001356663
D_0__Bacteria;D_1__Firmicutes;D_2__Clostridia;D_3__Clostridiales;D_4__Lachnospiraceae;D_5__Lachnospiraceae NK4A136 group	41	0.00041036	0.001384661
D_0__Bacteria;D_1__Firmicutes;D_2__Clostridia;D_3__Clostridiales;D_4__Lachnospiraceae;D_5__Lachnospiraceae ND3007 group	41	0.000403781	0.001808166
D_0__Bacteria;D_1__Proteobacteria;D_2__Gammaproteobacteria;D_3__Enterobacteriales;D_4__Enterobacteriaceae;D_5__Proteus	41	0.000392237	0.002511545
D_0__Bacteria;D_1__Firmicutes;D_2__Clostridia;D_3__Clostridiales;D_4__Ruminococcaceae;D_5__Flavonifractor	41	0.000387516	0.002100308
D_0__Bacteria;D_1__Firmicutes;D_2__Clostridia;D_3__Clostridiales;D_4__Lachnospiraceae;D_5__[Ruminococcus] torques group	41	0.000367368	0.001204808
D_0__Bacteria;D_1__Firmicutes;D_2__Negativicutes;D_3__Selenomonadales;D_4__Veillonellaceae;D_5__Megamonas	41	0.000365011	0.002337213
D_0__Bacteria;D_1__Proteobacteria;D_2__Gammaproteobacteria;D_3__Pasteurellales;D_4__Pasteurellaceae;Other	41	0.000362937	0.001374186
D_0__Bacteria;D_1__Firmicutes;D_2__Clostridia;D_3__Clostridiales;D_4__Lachnospiraceae;D_5__Fusicatenibacter	41	0.000362703	0.001152883
D_0__Bacteria;D_1__Actinobacteria;D_2__Actinobacteria;D_3__Corynebacteriales;D_4__Corynebacteriaceae;D_5__Corynebacterium 1	41	0.000337922	0.000769924
D_0__Bacteria;D_1__Fusobacteria;D_2__Fusobacteriia;D_3__Fusobacteriales;D_4__Fusobacteriaceae;D_5__Fusobacterium	41	0.000332878	0.000979205
D_0__Bacteria;D_1__Firmicutes;D_2__Clostridia;D_3__Clostridiales;D_4__Lachnospiraceae;D_5__[Eubacterium] hallii group	41	0.000327791	0.00106607

D_0__Bacteria;D_1__Proteobacteria;D_2__Gammaproteobacteria;D_3__Betaproteobacteriales;D_4__Burkholderiaceae;D_5__Parasutterella	41	0.000315052	0.001278827
D_0__Bacteria;D_1__Firmicutes;D_2__Clostridia;D_3__Clostridiales;D_4__Lachnospiraceae;D_5__uncultured	41	0.000315047	0.001935245
D_0__Bacteria;D_1__Actinobacteria;D_2__Coriobacteriia;D_3__Coriobacteriales;D_4__Eggerthellaceae;D_5__Gordonibacter	41	0.000306855	0.001568106
D_0__Bacteria;D_1__Firmicutes;D_2__Clostridia;D_3__Clostridiales;D_4__Lachnospiraceae;D_5__[Ruminococcus] gauvreauii group	41	0.000306169	0.001228499
D_0__Bacteria;D_1__Firmicutes;D_2__Clostridia;D_3__Clostridiales;D_4__Ruminococcaceae;D_5__Ruminococcaceae UCG-014	41	0.00029135	0.001115715
D_0__Bacteria;D_1__Firmicutes;D_2__Bacilli;D_3__Lactobacillales;D_4__Carnobacteriaceae;D_5__Dolosigranulum	41	0.000287202	0.001706829
D_0__Bacteria;D_1__Firmicutes;D_2__Negativicutes;D_3__Selenomonadales;D_4__Veillonellaceae;D_5__Megasphaera	41	0.000284413	0.001520869
D_0__Bacteria;D_1__Proteobacteria;D_2__Gammaproteobacteria;D_3__Enterobacteriales;D_4__Enterobacteriaceae;D_5__Morganella	41	0.000258139	0.001652897
D_0__Bacteria;D_1__Firmicutes;D_2__Clostridia;D_3__Clostridiales;D_4__Lachnospiraceae;D_5__[Eubacterium] eligens group	41	0.00025743	0.00136338
D_0__Bacteria;D_1__Firmicutes;D_2__Clostridia;D_3__Clostridiales;D_4__Ruminococcaceae;D_5__Ruminococcaceae UCG-002	41	0.000232843	0.000651681
D_0__Bacteria;D_1__Actinobacteria;D_2__Coriobacteriia;D_3__Coriobacteriales;D_4__Atopobiaceae;D_5__Atopobium	41	0.000230539	0.000788914
D_0__Bacteria;D_1__Firmicutes;D_2__Clostridia;D_3__Clostridiales;D_4__Lachnospiraceae;D_5__Tyzzerella 3	41	0.000220838	0.001031127
D_0__Bacteria;D_1__Firmicutes;D_2__Clostridia;D_3__Clostridiales;D_4__Lachnospiraceae;D_5__Lachnospiraceae UCG-008	41	0.000191129	0.001223826
D_0__Archaea;D_1__Euryarchaeota;D_2__Methanobacteria;D_3__Methanobacteriales;D_4__Methanobacteriaceae;D_5__Methanobrevibacter	41	0.000182395	0.000489421
D_0__Bacteria;D_1__Firmicutes;D_2__Clostridia;D_3__Clostridiales;D_4__Lachnospiraceae;D_5__Dorea	41	0.000177035	0.000826475
D_0__Bacteria;D_1__Firmicutes;D_2__Clostridia;D_3__Clostridiales;D_4__Lachnospiraceae;D_5__Moryella	41	0.000162729	0.001041976
D_0__Bacteria;D_1__Actinobacteria;D_2__Actinobacteria;D_3__Actinomycetales;D_4__Actinomycetaceae;D_5__Varibaculum	41	0.000155328	0.000690092
D_0__Bacteria;D_1__Bacteroidetes;D_2__Bacteroidia;D_3__Bacteroidales;D_4__Marinifilaceae;D_5__Butyricimonas	41	0.000142689	0.000549903
D_0__Bacteria;D_1__Firmicutes;D_2__Clostridia;D_3__Clostridiales;D_4__Ruminococcaceae;D_5__Ruminococcaceae UCG-013	41	0.000130491	0.000656814
D_0__Bacteria;D_1__Bacteroidetes;D_2__Bacteroidia;D_3__Bacteroidales;D_4__Rikenellaceae;D_5__Rikenellaceae RC9 gut group	41	0.00011484	0.000502584
D_0__Bacteria;D_1__Firmicutes;D_2__Clostridia;D_3__Clostridiales;D_4__Lachnospiraceae;D_5__Butyrivibrio	41	0.000100965	0.000646489
D_0__Bacteria;D_1__Actinobacteria;D_2__Actinobacteria;D_3__Bifidobacteriales;D_4__Bifidobacteriaceae;D_5__Scardovia	41	0.000100707	0.000567756
D_0__Bacteria;D_1__Firmicutes;D_2__Clostridia;D_3__Clostridiales;D_4__Ruminococcaceae;D_5__CAG-352	41	9.56706E-05	0.000432357
D_0__Bacteria;Other;Other;Other;Other;Other	41	9.31195E-05	0.000187799
D_0__Bacteria;D_1__Actinobacteria;D_2__Actinobacteria;D_3__Micrococcales;D_4__Micrococcaceae;Other	41	9.26905E-05	0.000433277
D_0__Bacteria;D_1__Firmicutes;D_2__Clostridia;D_3__Clostridiales;D_4__Ruminococcaceae;Other	41	8.47668E-05	0.000389333
D_0__Bacteria;D_1__Firmicutes;D_2__Clostridia;D_3__Clostridiales;D_4__Ruminococcaceae;D_5__Oscillibacter	41	8.41969E-05	0.000413423
D_0__Bacteria;D_1__Firmicutes;D_2__Clostridia;D_3__Clostridiales;D_4__Ruminococcaceae;D_5__[Eubacterium] coprostanoligenes group	41	8.04845E-05	0.00025304
D_0__Bacteria;D_1__Proteobacteria;D_2__Gammaproteobacteria;D_3__Enterobacteriales;D_4__Enterobacteriaceae;D_5__Hafnia-Obesumbacterium	41	8.01135E-05	0.000512977
D_0__Bacteria;D_1__Bacteroidetes;D_2__Bacteroidia;D_3__Bacteroidales;D_4__Prevotellaceae;D_5__Prevotella 7	41	7.69263E-05	0.000492569
D_0__Bacteria;D_1__Firmicutes;D_2__Clostridia;D_3__Clostridiales;D_4__Lachnospiraceae;D_5__Coprococcus 3	41	7.27987E-05	0.000339135
D_0__Bacteria;D_1__Proteobacteria;D_2__Alphaproteobacteria;D_3__Rhodospirillales;D_4__uncultured;D_5__Azospirillum sp, 47_25	41	6.82341E-05	0.000338625
D_0__Bacteria;D_1__Firmicutes;D_2__Clostridia;D_3__Clostridiales;D_4__Clostridiaceae 1;D_5__Clostridium sensu stricto 18	41	6.77883E-05	0.000434057
D_0__Bacteria;D_1__Firmicutes;D_2__Clostridia;D_3__Clostridiales;D_4__Lachnospiraceae;D_5__Lachnospiraceae UCG-001	41	6.76437E-05	0.000332609
D_0__Bacteria;D_1__Firmicutes;D_2__Clostridia;D_3__Clostridiales;D_4__Ruminococcaceae;D_5__Ruminiclostridium 9	41	6.65311E-05	0.000333222
D_0__Bacteria;D_1__Firmicutes;D_2__Bacilli;D_3__Lactobacillales;Other;Other	41	5.93131E-05	0.000270351
D_0__Bacteria;D_1__Firmicutes;D_2__Clostridia;D_3__Clostridiales;D_4__Lachnospiraceae;D_5__Coprococcus 2	41	5.75809E-05	0.000304991
D_0__Bacteria;D_1__Firmicutes;D_2__Clostridia;D_3__Clostridiales;D_4__Lachnospiraceae;D_5__[Eubacterium] fissicatena group	41	5.53067E-05	0.000354136
D_0__Bacteria;D_1__Proteobacteria;D_2__Gammaproteobacteria;D_3__Enterobacteriales;D_4__Enterobacteriaceae;D_5__Kluyvera	41	5.22389E-05	0.000334492
D_0__Bacteria;D_1__Proteobacteria;D_2__Gammaproteobacteria;Other;Other;Other	41	5.01172E-05	0.000188698
D_0__Bacteria;D_1__Bacteroidetes;D_2__Bacteroidia;D_3__Bacteroidales;D_4__Prevotellaceae;D_5__Paraprevotella	41	4.96414E-05	0.000281966
D_0__Bacteria;D_1__Firmicutes;D_2__Clostridia;D_3__Clostridiales;D_4__Peptostreptococcaceae;D_5__Romboutsia	41	4.8389E-05	0.000154891
D_0__Bacteria;D_1__Bacteroidetes;D_2__Bacteroidia;D_3__Bacteroidales;D_4__Barnesiellaceae;D_5__Barnesiella	41	4.82181E-05	0.000173327
D_0__Bacteria;D_1__Firmicutes;D_2__Clostridia;D_3__Clostridiales;D_4__Ruminococcaceae;D_5__Butyricicoccus	41	4.82181E-05	0.000184765
D_0__Bacteria;D_1__Firmicutes;D_2__Clostridia;D_3__Clostridiales;D_4__Christensenellaceae;D_5__Christensenellaceae R-7 group	41	4.34196E-05	0.000224727
D_0__Bacteria;D_1__Actinobacteria;D_2__Actinobacteria;D_3__Micrococcales;D_4__Dermabacteraceae;D_5__Dermabacter	41	4.18037E-05	0.000267675
D_0__Bacteria;D_1__Proteobacteria;D_2__Alphaproteobacteria;D_3__Rhodobacterales;D_4__Rhodobacteraceae;D_5__Paracoccus	41	3.55977E-05	0.000227937
D_0__Bacteria;D_1__Bacteroidetes;D_2__Bacteroidia;D_3__Bacteroidales;D_4__Prevotellaceae;D_5__Prevotella 6	41	3.49717E-05	0.000165636
D_0__Bacteria;D_1__Firmicutes;D_2__Negativicutes;D_3__Selenomonadales;D_4__Acidaminococcaceae;D_5__Phascolarctobacterium	41	3.27574E-05	0.00020975
D_0__Bacteria;D_1__Bacteroidetes;D_2__Bacteroidia;D_3__Bacteroidales;D_4__Muribaculaceae;D_5__uncultured bacterium	41	3.2386E-05	0.000198489
D_0__Bacteria;D_1__Firmicutes;D_2__Clostridia;D_3__Clostridiales;D_4__Clostridiaceae 1;Other	41	3.12841E-05	0.000200316
D_0__Bacteria;D_1__Firmicutes;D_2__Clostridia;D_3__Clostridiales;D_4__Lachnospiraceae;D_5__CAG-56	41	3.12812E-05	0.00018703
D_0__Bacteria;D_1__Firmicutes;D_2__Clostridia;D_3__Clostridiales;D_4__Ruminococcaceae;D_5__Intestinimonas	41	3.03066E-05	0.000177458
D_0__Bacteria;D_1__Actinobacteria;D_2__Actinobacteria;D_3__Actinomycetales;D_4__Actinomycetaceae;D_5__Actinobaculum	41	2.98803E-05	0.000191327
D_0__Bacteria;D_1__Firmicutes;D_2__Clostridia;D_3__Clostridiales;D_4__Lachnospiraceae;D_5__[Eubacterium] xylanophilum group	41	2.97805E-05	0.000190688
D_0__Bacteria;D_1__Cyanobacteria;D_2__Melainabacteria;D_3__Gastranaerophilales;Other;Other	41	2.97805E-05	0.000190688
D_0__Bacteria;D_1__Proteobacteria;D_2__Gammaproteobacteria;D_3__Pseudomonadales;D_4__Moraxellaceae;D_5__Acinetobacter	41	2.90133E-05	0.00013119
D_0__Bacteria;D_1__Firmicutes;D_2__Clostridia;D_3__Clostridiales;D_4__Ruminococcaceae;D_5__GCA-900066225	41	2.65897E-05	0.000170257
D_0__Bacteria;D_1__Firmicutes;D_2__Bacilli;D_3__Bacillales;D_4__Paenibacillaceae;D_5__Paenibacillus	41	2.52666E-05	0.000161785
D_0__Bacteria;D_1__Proteobacteria;D_2__Gammaproteobacteria;D_3__Betaproteobacteriales;D_4__Neisseriaceae;D_5__Neisseria	41	2.41472E-05	0.000120309
D_0__Bacteria;D_1__Firmicutes;D_2__Clostridia;D_3__Clostridiales;D_4__Ruminococcaceae;D_5__Ruminococcaceae NK4A214 group	41	2.38868E-05	0.000107062
D_0__Bacteria;D_1__Firmicutes;D_2__Clostridia;D_3__Clostridiales;D_4__Peptostreptococcaceae;D_5__Paeniclostridium	41	2.3399E-05	0.000149827
D_0__Bacteria;D_1__Firmicutes;D_2__Clostridia;D_3__Clostridiales;D_4__Lachnospiraceae;D_5__[Eubacterium] ventriosum group	41	2.3399E-05	0.000149827
D_0__Bacteria;D_1__Firmicutes;D_2__Clostridia;D_3__Clostridiales;D_4__Family XI;D_5__Finegoldia	41	2.22955E-05	9.86967E-05
D_0__Bacteria;D_1__Firmicutes;Other;Other;Other;Other	41	2.10318E-05	0.000127497
D_0__Bacteria;D_1__Firmicutes;D_2__Clostridia;D_3__Clostridiales;D_4__Ruminococcaceae;D_5__Ruminococcaceae UCG-010	41	2.09851E-05	8.33324E-05
D_0__Bacteria;D_1__Firmicutes;D_2__Clostridia;D_3__Clostridiales;D_4__Lachnospiraceae;D_5__Lachnospiraceae FCS020 group	41	2.09524E-05	9.59904E-05
D_0__Bacteria;D_1__Firmicutes;D_2__Clostridia;D_3__Clostridiales;D_4__Lachnospiraceae;D_5__Tyzzerella 4	41	1.99081E-05	0.000127474
D_0__Bacteria;D_1__Firmicutes;D_2__Clostridia;D_3__Clostridiales;D_4__Peptostreptococcaceae;Other	41	1.88202E-05	0.000112865
D_0__Bacteria;D_1__Bacteroidetes;D_2__Bacteroidia;D_3__Bacteroidales;D_4__Prevotellaceae;D_5__Prevotellaceae UCG-001	41	1.67363E-05	7.13022E-05
D_0__Bacteria;D_1__Bacteroidetes;D_2__Bacteroidia;D_3__Bacteroidales;D_4__Prevotellaceae;D_5__Alloprevotella	41	1.61858E-05	8.41249E-05
D_0__Bacteria;D_1__Firmicutes;D_2__Clostridia;D_3__Clostridiales;D_4__Family XI;D_5__Anaerococcus	41	1.52457E-05	8.41762E-05
D_0__Bacteria;D_1__Firmicutes;D_2__Clostridia;D_3__Clostridiales;D_4__Peptostreptococcaceae;D_5__Terrisporobacter	41	1.46239E-05	9.36383E-05
D_0__Bacteria;D_1__Firmicutes;D_2__Clostridia;D_3__Clostridiales;D_4__Lachnospiraceae;D_5__GCA-900066575	41	1.38267E-05	8.85339E-05
D_0__Bacteria;D_1__Firmicutes;D_2__Clostridia;D_3__Clostridiales;D_4__Lachnospiraceae;D_5__Lachnospiraceae UCG-004	41	1.37972E-05	8.83451E-05
D_0__Bacteria;D_1__Firmicutes;D_2__Clostridia;D_3__Clostridiales;D_4__Lachnospiraceae;D_5__Lachnoanaerobaculum	41	1.33594E-05	7.14138E-05
D_0__Bacteria;D_1__Firmicutes;D_2__Clostridia;D_3__Clostridiales;D_4__Ruminococcaceae;D_5__Ruminococcaceae UCG-005	41	1.27726E-05	8.17847E-05
D_0__Bacteria;D_1__Proteobacteria;D_2__Deltaproteobacteria;D_3__Desulfovibrionales;D_4__Desulfovibrionaceae;D_5__uncultured	41	1.27631E-05	8.17236E-05
D_0__Bacteria;D_1__Firmicutes;D_2__Clostridia;D_3__Clostridiales;D_4__Lachnospiraceae;D_5__[Eubacterium] ruminantium group	41	1.16991E-05	7.49107E-05
D_0__Bacteria;D_1__Firmicutes;D_2__Clostridia;D_3__Clostridiales;D_4__Ruminococcaceae;D_5__Ruminiclostridium 6	41	1.16294E-05	5.2715E-05
D_0__Bacteria;D_1__Firmicutes;D_2__Erysipelotrichia;D_3__Erysipelotrichales;D_4__Erysipelotrichaceae;D_5__Faecalitalea	41	1.1376E-05	7.28422E-05
D_0__Bacteria;D_1__Firmicutes;D_2__Clostridia;D_3__Clostridiales;D_4__Clostridiales vadinBB60 group;D_5__uncultured organism	41	9.8188E-06	4.9673E-05
D_0__Bacteria;D_1__Firmicutes;D_2__Bacilli;D_3__Lactobacillales;D_4__Streptococcaceae;D_5__Lactococcus	41	9.35926E-06	5.99285E-05
D_0__Bacteria;D_1__Bacteroidetes;D_2__Bacteroidia;D_3__Bacteroidales;D_4__Barnesiellaceae;D_5__uncultured	41	9.32884E-06	5.97337E-05
D_0__Bacteria;D_1__Proteobacteria;D_2__Gammaproteobacteria;D_3__Betaproteobacteriales;D_4__Burkholderiaceae;Other	41	8.89943E-06	5.69842E-05
D_0__Bacteria;D_1__Actinobacteria;D_2__Actinobacteria;Other;Other;Other	41	8.43898E-06	5.40359E-05
D_0__Bacteria;D_1__Tenericutes;D_2__Mollicutes;D_3__Izimaplasmatales;D_4__uncultured organism;D_5__	41	8.20699E-06	3.68202E-05
D_0__Bacteria;D_1__Firmicutes;D_2__Bacilli;D_3__Lactobacillales;D_4__Enterococcaceae;Other	41	8.12279E-06	4.20229E-05
D_0__Bacteria;D_1__Proteobacteria;D_2__Alphaproteobacteria;D_3__Rhizobiales;D_4__Rhizobiaceae;D_5__Allorhizobium-Neorhizobium-Pararhizobium-Rhizobium	41	7.3517E-06	4.70739E-05
D_0__Bacteria;D_1__Firmicutes;D_2__Clostridia;D_3__Clostridiales;D_4__Ruminococcaceae;D_5__Ruminococcaceae UCG-003	41	7.01945E-06	4.49464E-05
D_0__Bacteria;D_1__Bacteroidetes;D_2__Bacteroidia;D_3__Bacteroidales;D_4__Barnesiellaceae;D_5__Coprobacter	41	6.70491E-06	4.29324E-05
D_0__Bacteria;D_1__Proteobacteria;D_2__Gammaproteobacteria;D_3__Xanthomonadales;D_4__Xanthomonadaceae;D_5__Pseudoxanthomonas	41	5.80398E-06	3.71636E-05
D_0__Bacteria;D_1__Firmicutes;D_2__Clostridia;D_3__Clostridiales;D_4__Family XI;D_5__Peptoniphilus	41	5.77242E-06	2.79814E-05
D_0__Bacteria;D_1__Firmicutes;D_2__Erysipelotrichia;D_3__Erysipelotrichales;D_4__Erysipelotrichaceae;D_5__Holdemania	41	5.68802E-06	3.64211E-05
D_0__Bacteria;D_1__Actinobacteria;D_2__Coriobacteriia;D_3__Coriobacteriales;D_4__Eggerthellaceae;D_5__uncultured	41	5.68802E-06	3.64211E-05
D_0__Bacteria;D_1__Actinobacteria;D_2__Actinobacteria;D_3__Micrococcales;D_4__Dermatophilaceae;D_5__uncultured	41	5.66359E-06	3.62647E-05
D_0__Bacteria;D_1__Firmicutes;D_2__Clostridia;D_3__Clostridiales;D_4__Defluviitaleaceae;D_5__Defluviitaleaceae UCG-011	41	5.31795E-06	3.40515E-05
D_0__Bacteria;D_1__Firmicutes;D_2__Bacilli;D_3__Lactobacillales;D_4__Leuconostocaceae;D_5__Leuconostoc	41	5.31795E-06	3.40515E-05
D_0__Bacteria;D_1__Lentisphaerae;D_2__Lentisphaeria;D_3__Victivallales;D_4__vadinBE97;Other	41	4.75861E-06	3.047E-05
D_0__Bacteria;D_1__Firmicutes;D_2__Clostridia;D_3__Clostridiales;D_4__Family XI;D_5__Ezakiella	41	4.71329E-06	3.01798E-05
D_0__Bacteria;D_1__Actinobacteria;Other;Other;Other;Other	41	4.51995E-06	2.30023E-05
D_0__Bacteria;D_1__Proteobacteria;Other;Other;Other;Other	41	4.26642E-06	2.73184E-05
D_0__Bacteria;D_1__Proteobacteria;D_2__Gammaproteobacteria;D_3__Pseudomonadales;D_4__Pseudomonadaceae;D_5__Pseudomonas	41	4.25625E-06	2.72533E-05
D_0__Bacteria;D_1__Firmicutes;D_2__Clostridia;D_3__Clostridiales;Other;Other	41	4.25436E-06	2.72412E-05
D_0__Bacteria;D_1__Firmicutes;D_2__Clostridia;D_3__Clostridiales;D_4__Ruminococcaceae;D_5__Candidatus Soleaferrea	41	4.25436E-06	2.72412E-05
D_0__Bacteria;D_1__Cyanobacteria;D_2__Oxyphotobacteria;D_3__Chloroplast;Other;Other	41	3.78542E-06	2.42385E-05
D_0__Bacteria;D_1__Lentisphaerae;D_2__Lentisphaeria;D_3__Victivallales;D_4__Victivallaceae;D_5__Victivallis	41	3.64932E-06	2.33671E-05
D_0__Bacteria;D_1__Firmicutes;D_2__Clostridia;D_3__Clostridiales;D_4__Lachnospiraceae;D_5__Lachnoclostridium 12	41	3.64932E-06	2.33671E-05
D_0__Bacteria;D_1__Firmicutes;D_2__Clostridia;D_3__Clostridiales;D_4__Lachnospiraceae;D_5__Howardella	41	3.50972E-06	2.24732E-05
D_0__Bacteria;D_1__Bacteroidetes;D_2__Bacteroidia;D_3__Bacteroidales;D_4__Prevotellaceae;D_5__Prevotellaceae NK3B31 group	41	3.50972E-06	2.24732E-05
D_0__Bacteria;D_1__Proteobacteria;D_2__Alphaproteobacteria;D_3__Rhodospirillales;D_4__uncultured;Other	41	3.49104E-06	1.56116E-05
D_0__Bacteria;D_1__Elusimicrobia;D_2__Elusimicrobia;D_3__Elusimicrobiales;D_4__Elusimicrobiaceae;D_5__Elusimicrobium	41	3.41313E-06	2.18547E-05
D_0__Bacteria;D_1__Actinobacteria;D_2__Actinobacteria;D_3__Corynebacteriales;D_4__Corynebacteriaceae;D_5__Lawsonella	41	3.18397E-06	2.03873E-05
D_0__Bacteria;D_1__Actinobacteria;D_2__Coriobacteriia;D_3__Coriobacteriales;D_4__Eggerthellaceae;D_5__Adlercreutzia	41	2.65331E-06	1.69894E-05

D_0__Bacteria;D_1__Firmicutes;D_2__Clostridia;D_3__Clostridiales;D_4__Peptostreptococcaceae;D_5__Asaccharospora	41	2.6097E-06	1.67102E-05
D_0__Bacteria;D_1__Firmicutes;D_2__Erysipelotrichia;D_3__Erysipelotrichales;D_4__Erysipelotrichaceae;D_5__Solobacterium	41	2.12718E-06	1.36206E-05
D_0__Bacteria;D_1__Bacteroidetes;D_2__Bacteroidia;D_3__Bacteroidales;D_4__Muribaculaceae;D_5__metagenome	41	1.70657E-06	1.09274E-05
D_0__Archaea;D_1__Euryarchaeota;D_2__Thermoplasmata;D_3__Methanomassiliicoccales;D_4__Methanomethylophilaceae;D_5__uncultured	41	1.70657E-06	1.09274E-05
D_0__Bacteria;D_1__Actinobacteria;D_2__Coriobacteriia;D_3__Coriobacteriales;D_4__Coriobacteriales Incertae Sedis;D_5__uncultured	41	1.68561E-06	1.07932E-05
D_0__Bacteria;D_1__Firmicutes;D_2__Clostridia;D_3__Clostridiales;D_4__Lachnospiraceae;D_5__Lachnospiraceae NK4B4 group	41	1.59198E-06	1.01937E-05
D_0__Bacteria;D_1__Bacteroidetes;D_2__Bacteroidia;D_3__Flavobacteriales;D_4__Weeksellaceae;D_5__Chryseobacterium	41	1.54773E-06	9.91029E-06
D_0__Bacteria;D_1__Proteobacteria;D_2__Gammaproteobacteria;D_3__Pseudomonadales;D_4__Moraxellaceae;D_5__Enhydrobacter	41	1.20472E-06	7.714E-06
D_0__Bacteria;D_1__Bacteroidetes;D_2__Bacteroidia;D_3__Flavobacteriales;D_4__Flavobacteriaceae;D_5__uncultured	41	1.16991E-06	7.49107E-06
D_0__Bacteria;D_1__Proteobacteria;D_2__Deltaproteobacteria;D_3__Desulfovibrionales;D_4__Desulfovibrionaceae;D_5__Desulfovibrio	41	1.1608E-06	7.43272E-06
D_0__Bacteria;D_1__Firmicutes;D_2__Clostridia;D_3__Clostridiales;D_4__Ruminococcaceae;D_5__Ruminiclostridium	41	0	0
D_0__Bacteria;D_1__Firmicutes;D_2__Bacilli;Other;Other;Other	41	0	0

online resource 4a

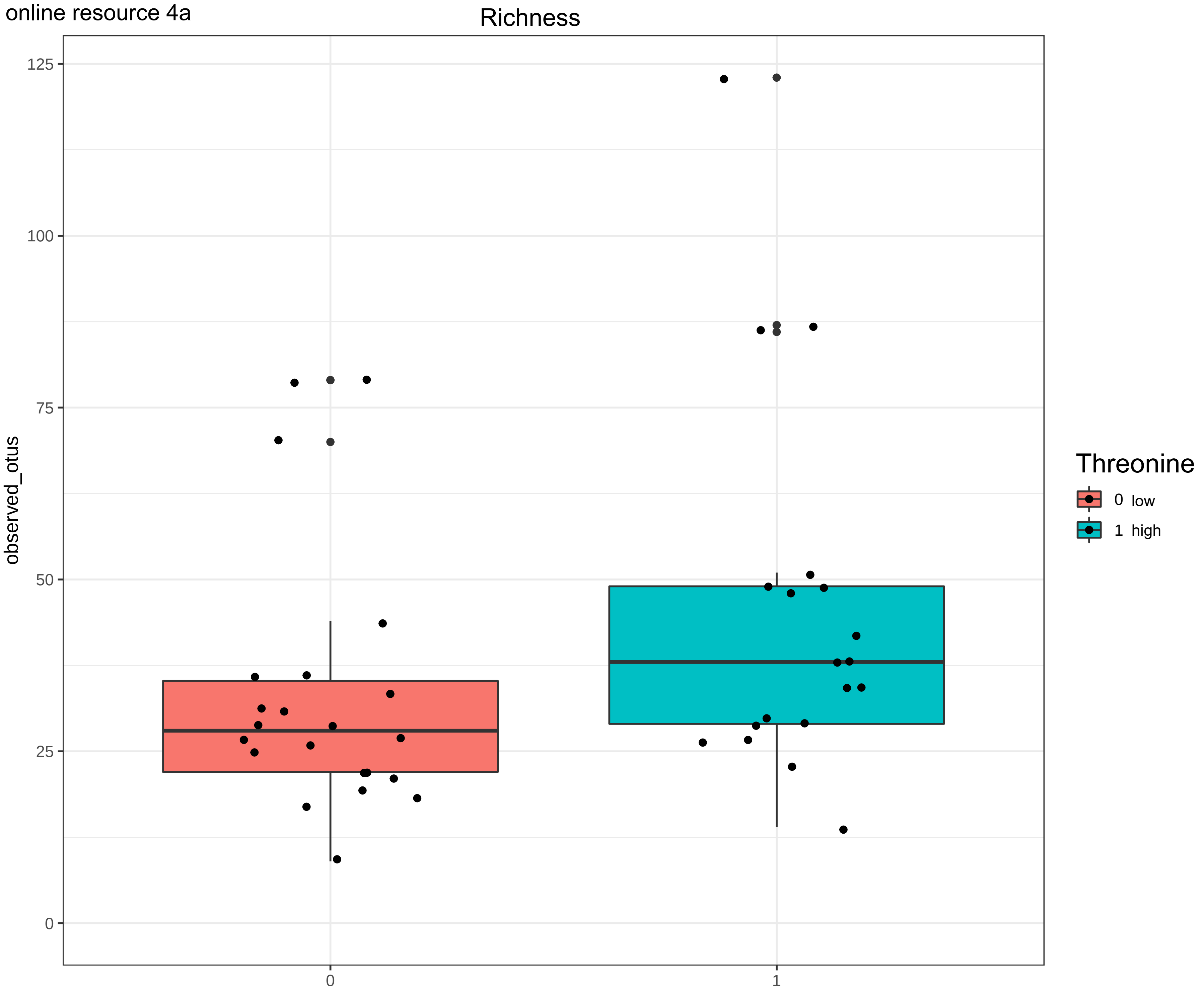

online resource 4b

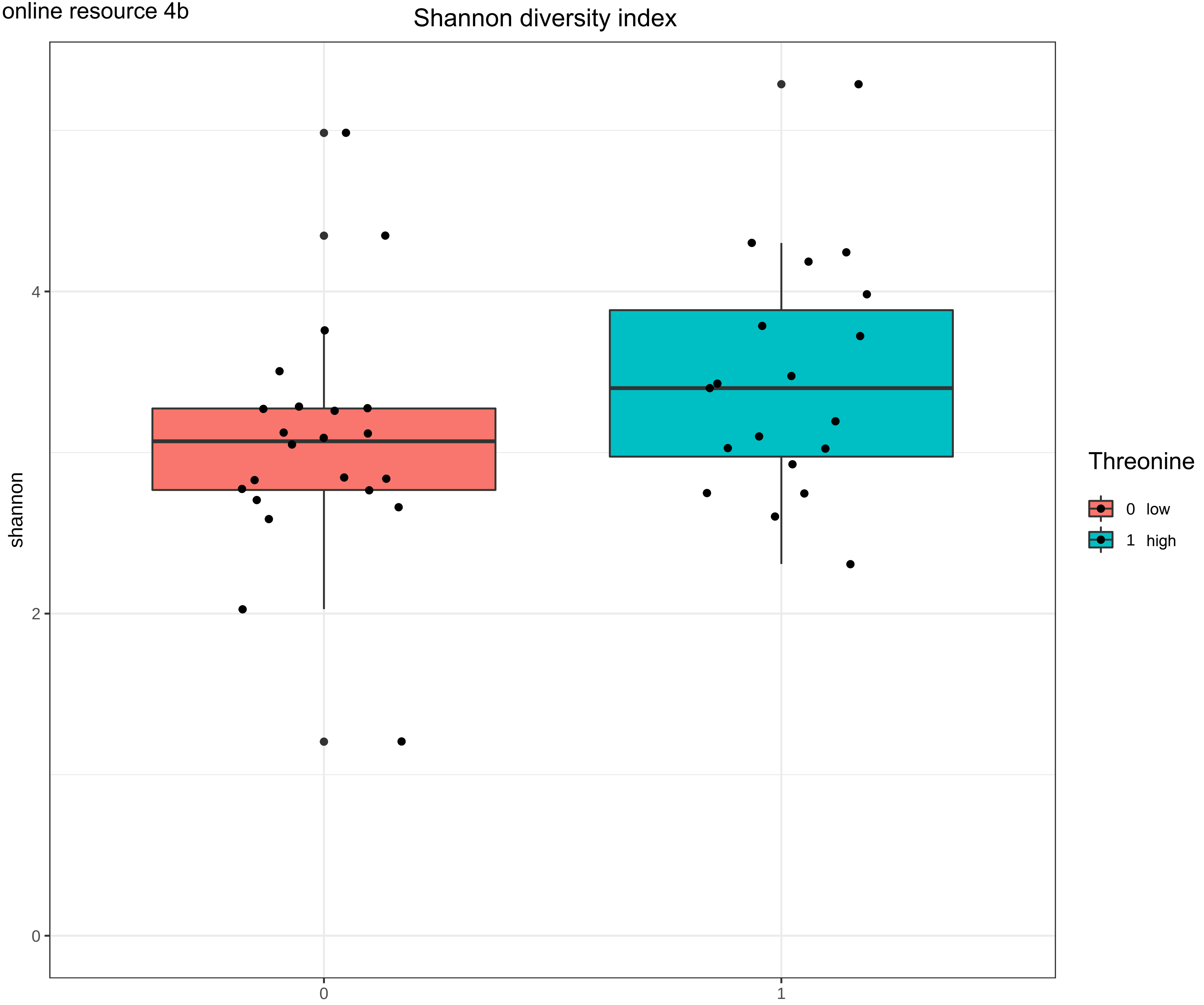

online resource 4c

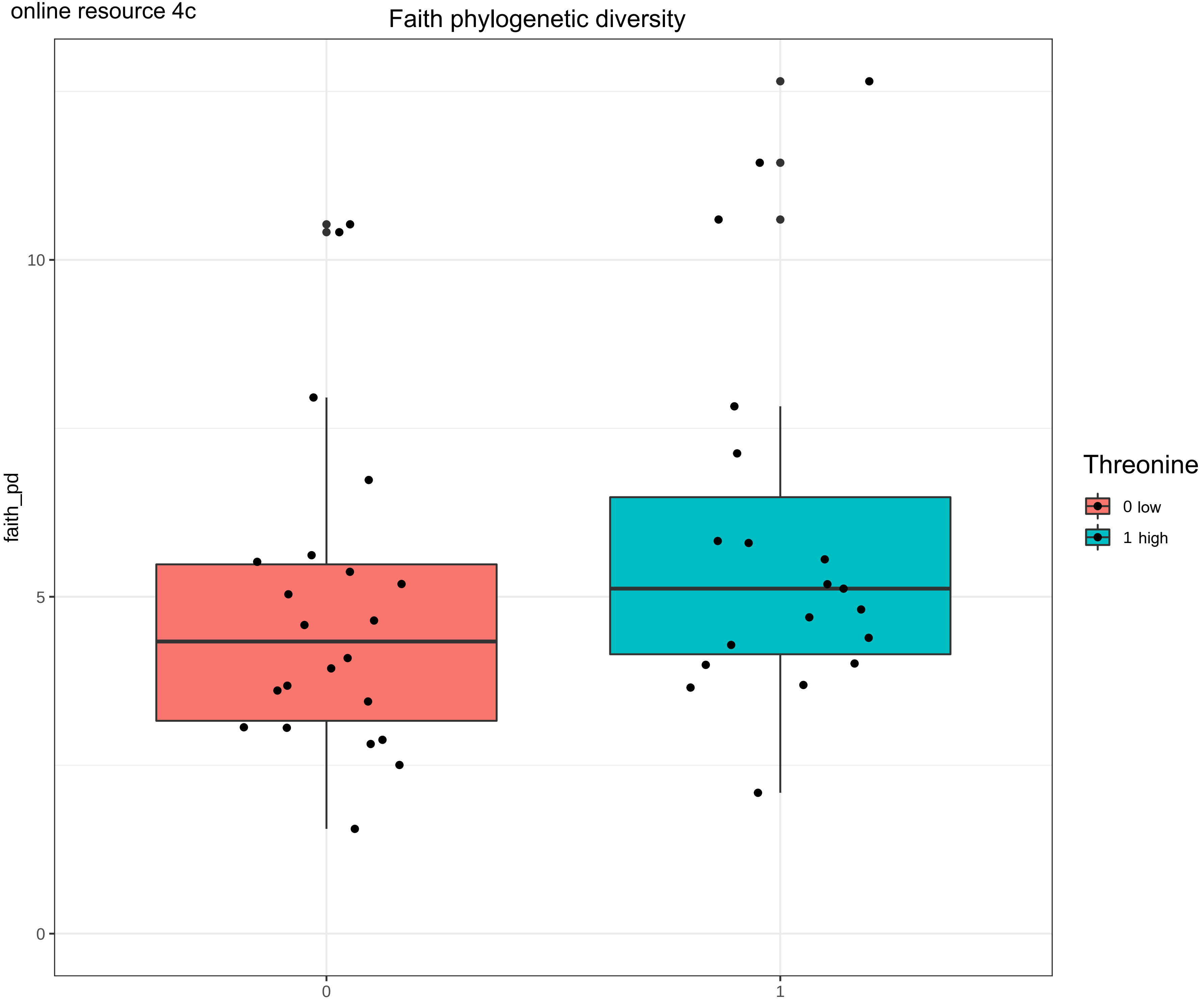

online resource 5: Linear regression analysis of human milk free threonine (in %) with bacterial entities adjusted for confounders [(a)] versus entities adjusted for confounders including exclusively breast fed category [(b)] [c]

Regression models	beta [(a)]	lower bound 95% CI [(a)]	upper bound 95% CI [(a)]	P-value [(a)]	R^2 (%) [(a)]	**adj. R^2 (%) [(a)]**	n	beta [(b)]	**p [(b)]**	**adj. R^2 (%) [(b)]**
**Proteobacteria (Phyla)**										
constant		0.52198454	3.14416293	0.0074983						
**Threonine LOG**	-0.441	-7.36806169	-1.30784836	0.006	**19.4**	**17.1**	37	-0.442	**0.006**	**17.3**
**Gammaproteobacteria (Class)**										
constant		0.75225573	3.30276894	0.00271039						
**Threonine LOG**	-0.48769792	-7.74538513	-1.85079985	0.00220009	**23.8**	**21.6**	37	-0.484	**0.002**	**21.2**
**Enterobacteriales/Enterbacteriaceae (Order/Fam)**										
constant		0.83131038	3.3554156	0.00185659						
**Threonine LOG**	-0.50353764	-7.87070397	-2.03715116	0.00148806	**25.4**	**23.2**	37	-0.515	**0.001**	**24.4**
**Escherichia_Shigella (Genus)**										
constant		0.17203068	2.88843006	0.02834069						
**Threonine LOG**	-0.36808169	-6.76026398	-0.48229303	0.02499672	**13.6**	**11.1**	37	-0.392	**0.016**	**12.9**

[c]Predicting amino acids were determined breast milk ; only significant results are shown ($p < 0.05$), beta = standardized regression coeffient, CI=Confidence Interval, R^2=coefficient of determination, adj R^2 = adjusted coefficient of determination, n=numbers of included participants (n is reduced as not all the parameters including confounders could be determined in every mother infant pair); **confounders (a):** GWG, parity, feeding mode (predominantly breast fed versus rest), birth mode, infant sex; **confounders (b):** GWG, parity, feeding mode (exclusively breast fed versus rest), birth mode, infant sex;
